# Optimal designs for phase II/III drug development programs including methods for discounting of phase II results

**DOI:** 10.1186/s12874-020-01093-w

**Published:** 2020-10-09

**Authors:** Stella Erdmann, Marietta Kirchner, Heiko Götte, Meinhard Kieser

**Affiliations:** 1 Institute of Medical Biometry and Informatics, University of Heidelberg, Im Neuenheimer Feld 130.3, D-69120 Heidelberg, Germany; 2 Merck Healthcare KGaA, Frankfurter Str. 250, D-64293 Darmstadt, Germany

**Keywords:** Optimization, Drug development program, Bias adjustment, Assurance, Probability of success, Sample size, Software

## Abstract

**Background:**

Go/no-go decisions after phase II and sample size chosen for phase III are usually based on phase II results (e.g., the treatment effect estimate of phase II). Due to the decision rule (only promising phase II results lead to phase III), treatment effect estimates from phase II that initiate a phase III trial commonly overestimate the true treatment effect. Underpowered phase III trials are the consequence. Optimistic findings may then not be reproduced, leading to the failure of potentially expensive drug development programs. For some disease areas these failure rates are described to be quite high: 62.5%.

**Methods:**

We integrate the ideas of multiplicative and additive adjustment of treatment effect estimates after go decisions in a utility-based framework for optimizing drug development programs. The design of a phase II/III program, i.e., the “right amount of adjustment”, the allocation of the resources to phase II and III in terms of sample size, and the rule applied to decide whether to stop or to proceed with phase III influences its success considerably. Given specific drug development program characteristics (e.g., fixed and variable per patient costs for phase II and III, probable gain in case of market launch), optimal designs with respect to the maximal expected utility can be identified by the proposed Bayesian-frequentist approach. The method will be illustrated by application to practical examples characteristic for oncological studies.

**Results:**

In general, our results show that the program set-ups with adjusted treatment effect estimate used for phase III planning are superior to the “naïve” program set-ups with respect to the maximal expected utility. Therefore, we recommend considering an adjusted phase II treatment effect estimate for the phase III sample size calculation. However, there is no one-fits-all design.

**Conclusion:**

Individual drug development planning for a specific program is necessary to find the optimal design. The optimal choice of the design parameters for a specific drug development program at hand can be found by our user friendly R Shiny application and package (both assessable open-source via [1]).

## Background

Exploratory studies are usually carried out to provide a basis for deciding whether or not to proceed with a confirmatory trial and, if necessary, to provide information for planning purposes. In drug development programs, this strong link between exploratory (e.g., phase II) and confirmatory (e.g., phase III) studies favors integrated planning. In particular, the costs of phase III studies have increased remarkably in recent years [2, 3], while failure rates are quite high (approx. 45%, see [4] and the reference mentioned therein). Therefore, the availability and application of quantitative methods for decision making, which should be data-driven and objective, is desirable [5].

Already over 30 years ago, Hughes and Pocock [6] pointed out that decision rules in clinical trials can lead to a bias in the point estimate of the treatment effect, so that the true underlying effect might be overestimated at the time of an early positive decision. Twenty four years and various attempts of authors to adjust for overestimation of the treatment effect (in group sequential designs) later (e.g., [7] and references mentioned therein), Zhang et al. [8] still criticize that the cause and effect of this phenomenon is generally not well-understood. Trying to illustrate the problem, they provide a graphical explanation for the occurrence of overestimation. They argue that random variability (i.e., random highs and lows) of the treatment effect estimate is always present, but stabilizes around the true treatment effect as the trial continues to its end. However, when implementing a decision rule the variability favors the random highs: in a phase II/III drug development program with a go/no-go decision rule, it is only proceeded with phase III when large treatment effects are observed, but stopped when small effects occur. This selective handling of random variability may lead to overestimation of the magnitude of the treatment effect after phase II.

Ellenberg et al. [9] as well as Nardini [10] emphasize that the aim of treatment effect estimation is not to decide whether or not one therapy is better than the other, but to describe the size of therapeutic effects. Thus, we are concerned with a problem of estimation, not a problem of testing. Nardini concludes that estimates arising after a decision rule “should [consequently] not be taken at face value as true estimates of the new treatment’s effect”. Ellenberg et al. point out that statistical methods to adjust for this “random-high bias” exist, but criticize that “they are not applied as often as they should be”. Recently, the U.S. Food & Drug Administration reported 22 case studies since 1999 in which promising phase II clinical trial results were not confirmed in phase III clinical testing [11]. Such experiences are not rare: for some disease areas, the failure rate for phase III trials is reported to be as high as 62.5% [12] and about 50% for approval [13]. Chuang-Stein and Kirby [14] give cause for serious concern, as the severity of this may multiply, considering that the bigger the estimated effect from, e.g., a proof of concept trial, the greater the temptation to invest heavily and conduct multiple studies in parallel. They advise to use the concept of “assurance” for quantification of success probabilities and, moreover, to apply an adjustment for the overestimation of the treatment effect (e.g., [15]) when planning the next phase of a drug development program.

In our framework, we follow the concept of “assurance” [16, 17], which had first been introduced by Spiegelhalter et al. in 1986 with the concept of Bayesian predictive power (compare also “average power”) [18, 19]. This methodology was used later in various contexts by O’Hagan et al. [16, 17] (“assurance”), Chuang-Stein [20], Chuang-Stein and Yang [21] (“average success probability”) and finally by Gasparini et al. and Saint-Hilary et al. (“predictive probability of success”) [22, 23]. The idea is to use a prior distribution for the true assumed treatment effect for trial planning. This is in contrast to the “frequentist world”, where a fixed value is assumed. The “assurance” is then the weighted (unconditional) probability of a successful trial for a given effect, the weighting resulting from the likelihood that the therapy will achieve this effect. Due to synthesizing Bayesian principles in the planning phase and frequentistic decision-making procedures in the analysis, the above-mentioned approaches are described in the literature as “mixed Bayesian-frequentist”.

Kirby et al. [15] and Wang et al. [24] attempt to reduce the impact of overestimation by discounting the phase II treatment effect estimate by applying a multiplicative or additive adjustment, respectively. However, their suggestions are not universally applicable, and are rather “rules of thumb”, e.g., Kirby et al. suggest to use a retention factor of 0.9 times the assumed ratio of the phase III effect to phase II effect.

De Martini [4, 25] reports that the phase II sample size should be almost as large as the ideal phase III sample size (at least 2/3 of the latter) in order to have a sufficiently good information basis for phase III planning. He criticizes that in practice this ratio is only 1/4 on average and that an increase in sponsorship gains from drug development through larger phase II studies has not yet been well investigated. Larger phase II sample sizes would reduce the level of overestimation but increase the estimated phase III sample size [26] and could retrospectively be regarded as an unnecessary high investment in case of a no-go decision. Therefore, an optimal balance is required.

In this article, we integrate the general concepts of using a multiplicative or additive adjustment method to correct for overestimation of the treatment effect in a framework of utility-based optimization of phase II/III development programs [27]. That is, we want to critically examine adjustment methods from an economic point of view. In addition to simultaneously optimizing the phase II go/no-go decision rule and the sample size, we also optimize over the adjustment parameter used for the phase II treatment effect estimation to find “the right level of adjustment” for the specific situation at hand. Our approach can build the bridge between the long existing gap of theory and practice: we provide a Bayesian-frequentist hybrid framework, in which methods proposed for addressing the problem of overestimation of the treatment effect after go decisions are included in the optimization of drug development programs.

In the second section of this paper, we will introduce the basic setting and notation, explain the adjustment methods and show how they are incorporated in our optimization framework. After introducing the utility function and explaining the optimization procedure, we present optimal designs for exemplary settings of drug development programs in Section 3. We finish with a discussion in Section 4 and a conclusion in Section 5.

## Methods

### Basic setting

**Fig. 1:**
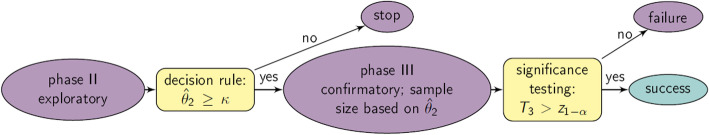
Graphical illustration of basic phase II/III drug development program. The drug development program consists of one exploratory phase II trial, which is, in case of a go decision (i.e., treatment effect estimate of phase II ${\hat{\theta}}_2$ exceeds predefined threshold value *κ* =  − log(*HR*_*go*_)), followed by one confirmatory phase III trial, where the sample size planning is based on ${\hat{\theta}}_2$. The program is considered successful if phase III has a positive (significant) result (i.e., normalized log rank test statistic of phase III *T*_3_ is above the 1 − *α* quantile of the standard normal distribution *z*_1 − *α*_)

The considered drug development program consists of one exploratory phase II and one confirmatory phase III trial. Both are randomized trials with two arms (each with 1:1 sample size allocation), performed independently, investigating the same time-to-event primary endpoint and the same population. The true treatment effect is given by the negative logarithm of the true hazard ratio (*θ* =  − log(*HR*)), which is the ratio of the hazard functions of the treatment and the control group. In order to reflect the uncertainty in the true treatment effect, *θ* can be modelled by a prior distribution *f*(*θ*). In phase II, the total number of events is denoted by *d*_2_ and the maximum likelihood estimate of *θ* is given by ${\hat{\theta}}_2$. We assume that the estimator ${\hat{\theta}}_2$ is asymptotically normally distributed with ${\hat{\theta}}_2\mid \theta \sim N\left(\theta, 4/{d}_2\right)$ (Note that the notation used will not differentiate between the treatment effect estimator (i.e., rule applied to estimate the quantity of interest, which is a random variable) and the treatment effect estimate (i.e., particular realization, fixed value), but by context it will be clear which quantity is meant.). Furthermore, we require that only phase II trials with promising results lead to a phase III trial. This is quantified by a go/no-go criterion with a go-decision in case of ${\hat{\theta}}_2\ge \kappa$, where *κ* is a predefined threshold value. In case of a go decision, the number of events for the phase III trial is calculated based on the observed treatment effect of phase II. If the confirmatory analysis in phase III reveals a significant result, program success is declared (compare Fig. 1).

Due to the decision rule after phase II, the treatment effect estimate of phase II is biased. The bias is positive with *κ* > 0 as probability mass is shifted towards higher values:

$$ {\displaystyle \begin{array}{l}E\left[{\hat{\theta}}_2|{\hat{\theta}}_2\ge \kappa \right]=\underset{-\infty }{\overset{\infty }{\int }}\underset{-\infty }{\overset{\infty }{\int }}{1}_{\left\{{\hat{\theta}}_2\ge \kappa \right\}}\cdot {\hat{\theta}}_2\cdot \frac{f\left({\hat{\theta}}_2|\theta \right)}{P\left({\hat{\theta}}_2\ge \kappa |\theta \right)}d{\hat{\theta}}_2\cdot f\left(\theta \right) d\theta \\ {}\kern6em +\underset{-\infty }{\overset{\infty }{\int }}\underset{-\infty }{\overset{\infty }{\int }}{1}_{\left\{{\hat{\theta}}_2\ge \kappa \right\}}\cdot {\hat{\theta}}_2\cdot 0\;d{\hat{\theta}}_2\cdot f\left(\theta \right) d\theta \\ {}\kern6em >\underset{-\infty }{\overset{\infty }{\int }}\underset{-\infty }{\overset{\infty }{\int }}{1}_{\left\{{\hat{\theta}}_2\ge \kappa \right\}}\cdot {\hat{\theta}}_2\cdot f\left({\hat{\theta}}_2|\theta \right)d{\hat{\theta}}_2\cdot f\left(\theta \right) d\theta \\ {}\kern6em +\underset{-\infty }{\overset{\infty }{\int }}\underset{-\infty }{\overset{\infty }{\int }}{1}_{\left\{{\hat{\theta}}_2\ge \kappa \right\}}\cdot {\hat{\theta}}_2\cdot f\left({\hat{\theta}}_2|\theta \right)d{\hat{\theta}}_2\cdot f\left(\theta \right) d\theta =\underset{-\infty }{\overset{\infty }{\int }}\underset{-\infty }{\overset{\infty }{\int }}{\hat{\theta}}_2\cdot f\left({\hat{\theta}}_2|\theta \right)\cdot f\left(\theta \right)d{\hat{\theta}}_2 d\theta =E\left[{\hat{\theta}}_2\right],\end{array}} $$

where here and in the following 1_*A*_ denotes the indicator function of event *A* and the density of the distribution of the respective argument is indicated by *f*(.). The inequation holds as $\frac{1}{P\left({\hat{\theta}}_2\ge \kappa |\theta \right)}>1$ and ${\int}_{-\infty}^{\infty }{1}_{\left\{{\hat{\theta}}_2\ge \kappa \right\}}\frac{f\left({\hat{\theta}}_2|\theta \right)}{P\left({\hat{\theta}}_2\ge \kappa |\theta \right)}d{\hat{\theta}}_2=1$ and, therefore, the probability mass assigned to values less than *κ* in the unconditional expectation $E\left[{\hat{\theta}}_2\right]$ is distributed between values greater than *κ* in $E\left[{\hat{\theta}}_2|{\hat{\theta}}_2\ge \kappa \right]$.

Note that the representation of the bias cannot be further simplified, neither by calculating $\mathrm{E}\left[{\hat{\theta}}_2\right]-\mathrm{E}\left[{\hat{\theta}}_2|{\hat{\theta}}_2\ge \kappa \right]$ nor $\mathrm{E}\left[{\hat{\theta}}_2\right]/\mathrm{E}\left[{\hat{\theta}}_2|{\hat{\theta}}_2\ge \kappa \right]$.

Therefore, in the following, multiplicative and additive adjustment methods for the treatment effect estimate obtained in phase II will be investigated. Afterwards, dependent on the respective adjustment method, launch criteria and approaches to calculate the number of events for phase III will be presented.

### Additive and multiplicative adjustment methods

In this section, we introduce two methods (an additive and a multiplicative adjustment method) to adjust for the overestimation of the phase II treatment effect estimate. It should be mentioned that the terms “multiplicative” and “additive” relate to the specific type of scale and endpoint considered here.

Wang et al. [24] advise to apply an additive adjustment to the phase II treatment effect estimate if it is used for planning the sample size of phase III. They discuss using the lower limit of the one and two standard deviation confidence interval (CI) from the phase II trial (i.e., the lower limit of the CI for ${\hat{\theta}}_2$, corresponding to one or two standard deviations below the point estimate), respectively. We denote the significance level of the lower bound for the one-sided CI related to the phase II treatment effect estimate as *α*_*CI*_ ∈ [0.025, 0.5] and define the additive adjusted treatment effect estimate by ${\hat{\theta}}_2^{a_{CI}}={\hat{\theta}}_2-{z}_{1-{a}_{CI}}\cdot \sqrt{4/{d}_2}$, with *z*_1 − *γ*_ = *Φ*^−1^(1 − *γ*), where *Φ*(.) denotes the distribution function of the standard normal distribution. Note that our version of the additive adjusted treatment effect estimate is a generalization of that of Wang et al., as they use the lower limit of the one and two standard deviation two-sided CI (i.e., in our notation *α*_*CI*_ = 0.32/2 and *α*_*CI*_ = 0.05/2) and we allow *α*_*CI*_ ranging from 0.025 to 0.5. For *α*_*CI*_ = 0.5, the additive adjusted treatment effect estimate is not discounted as ${\hat{\theta}}_2-{z}_{1-0.5}\cdot \sqrt{4/{d}_2}={\hat{\theta}}_2$.

Kirby et al. [15] propose a multiplicative adjustment approach. They multiply the observed treatment effect estimate with a factor *λ*, which can be understood as a retention factor, that is, the fraction of the treatment effect retained. Integrated in our setting, we define ${\hat{\theta}}_2^{\lambda }=\lambda \cdot {\hat{\theta}}_2$, where the multiplicative adjustment parameter *λ* ∈ [0.2, 1] can be viewed as the result of discounting the observed treatment effect of phase II by 1 − *λ*. Note that for *λ* = 1 the multiplicative adjusted treatment effect estimate is not discounted.

### Go/no-go criteria, calculation of expected number of events for phase III and related program characteristics

When designing the phase II/III program, the observed treatment effect estimate of phase II plays a key role in two ways: 1. when making the go/no-go decision (selection *s*_1_); 2. when calculating the phase III sample size (selection *s*_2_; compare Fig. 1). At both instances, one has to decide whether or not to use an adjusted or unadjusted treatment effect estimate. To ease notation, the naïve (unadjusted) treatment effect estimate of phase II is denoted by ${\hat{\theta}}_2^u={\hat{\theta}}_2$.

1.: If the treatment effect estimate ${\hat{\theta}}_2^{s_1}$, where *s*_1_ = *λ*, *α*_*CI*_ or *u* (i.e., the multiplicatively adjusted, additively adjusted or unadjusted treatment effect estimate is selected for the decision rule), exceeds a predefined threshold value *κ*, it is decided to go to phase III and otherwise to stop the program. Hence, the expected probability to go to phase III can be determined by

$$ {p}_{go}\left({\hat{\theta}}_2^{s_1}\right)={\int}_{-\infty}^{\infty }P\left({\hat{\theta}}_2^{s_1}\ge \kappa |\theta \right)\cdot f\left(\theta \right) d\theta, $$

*s*_1_ = *λ*, *α*_*CI*_ or *u* (compare Table A0 in the Additional file 1).

2.: In case of a go decision, the number of events for phase III is calculated based on the treatment effect estimate of phase II ${\hat{\theta}}_2^{s_2}$, *s*_2_ = *λ*, *α*_*CI*_ or *u*, a desired power 1 − *β*, and a one-sided significance level *α*. For a balanced allocation ratio, it can be calculated by

$$ {D}_3={D}_3\left({\hat{\theta}}_2^{s_2}\right)=\frac{4\cdot {\left({z}_{1-\alpha }+{z}_{1-\beta}\right)}^2}{{\left({\hat{\theta}}_2^{s_2}\right)}^2}, $$

by assuming proportional hazards and asymptotic properties of the log-rank test statistic [28]. When going to phase III, the expected number of events (in phase II/III programs with decision rule ${\hat{\theta}}_2^{s_1}\ge \kappa$ and ${\hat{\theta}}_2^{s_2}$ used to calculate the number of events for phase III) can be determined by

$$ {d}_3\left({\hat{\theta}}_2^{s_1},{\hat{\theta}}_2^{s_2}\right)=\mathrm{E}\left[{D}_3\left({\hat{\theta}}_2^{s_2}\right)\cdot {1}_{\left\{{\hat{\theta}}_2^{s_1}\ge \kappa \right\}}\right]={\int}_{-\infty}^{\infty }{\int}_{-\infty}^{\infty }{1}_{\left\{{\hat{\theta}}_2^{s_1}\ge \kappa \right\}}\cdot \frac{4\cdot {\left({z}_{1-\alpha }+{z}_{1-\beta}\right)}^2}{{\left({\hat{\theta}}_2^{s_2}\right)}^2}\cdot f\left({\hat{\theta}}_2|\theta \right)d{\hat{\theta}}_2\cdot f\left(\theta \right) d\theta, $$

(compare Table A0). The expectation of the estimate (of phase II) used for the sample size calculation can be calculated by

$$ {e}_2={e}_2\left({\hat{\theta}}_2^{s_1},{\hat{\theta}}_2^{s_2}\right)=\mathrm{E}\left[{\hat{\theta}}_2^{s_2}|{\hat{\theta}}_2^{s_1}\ge \kappa \right]={\int}_{-\infty}^{\infty}\frac{1}{\mathrm{P}\left({\hat{\theta}}_2^{s_1}\ge \kappa |\theta \right)}{\int}_{-\infty}^{\infty }{1}_{\left\{{\hat{\theta}}_2^{s_1}\ge \kappa \right\}}\cdot {\hat{\theta}}_2^{s_2}\cdot f\left({\hat{\theta}}_2|\theta \right)d{\hat{\theta}}_2\cdot f\left(\theta \right) d\theta, $$

for *s*_1_, *s*_2_ = *λ*, *α*_*CI*_ or *u* (compare Table A0) in order to calculate the bias $\mathrm{E}\left[{\hat{\theta}}_2^{s_2}|{\hat{\theta}}_2^{s_1}\ge \kappa \right]-\mathrm{E}\left[{\hat{\theta}}_2\right]$. As proposed by De Martini [4, 25], the ratio of the number of events in phase II and III will also be calculated.

The program is considered to be successful, if the one-sided null hypothesis *H*_0_ : *θ* ≤ 0 is rejected in favour of *H*_1_ : *θ* > 0 at a one-sided significance level *α*. This is the case if *T*_3_ > *z*_1 − *α*_, where *T*_3_ is the normalized log-rank test statistic in phase III, which is assumed to be asymptotically normally distributed, i.e., ${T}_3={T}_3\mid {\hat{\theta}}_2,\theta \sim N\left(\theta /\sqrt{4/{D}_3},1\right)$. Note that significance testing is performed on phase III data only. Therefore, the expected probability of a successful program $PsP\left({\hat{\theta}}_2^{s_1},{\hat{\theta}}_2^{s_2}\right)$ (with decision rule ${\hat{\theta}}_2^{s_1}\ge \kappa$, and ${\hat{\theta}}_2^{s_2}$ used to calculate the number of events for phase III), which is defined as the expected probability of the joint event of going to phase III and achieving a significant result [25, 27], can be calculated by

$$ PsP\left({\hat{\theta}}_2^{s_1},{\hat{\theta}}_2^{s_2}\right)={\int}_{-\infty}^{\infty }{\int}_{-\infty}^{\infty }{1}_{\left\{{\hat{\theta}}_2^{s_1}\ge \kappa \right\}}\cdot {\int}_{\left\{{z}_{1-\alpha}\right\}}^{\infty }f\left({t}_3|{\hat{\theta}}_2,\theta \right)d{t}_3\cdot f\left({\hat{\theta}}_2|\theta \right)d{\hat{\theta}}_2\cdot f\left(\theta \right) d\theta, $$

where *t*_3_ is a realization of ${T}_3\mid {\hat{\theta}}_2,\theta$ (compare Table A0). One reviewer pointed out that this definition of a successful program records a false positive result (i.e. *T*_3_ > *z*_1 − *α*_ under *H*_0_) as program success. We discuss this aspect in detail in Section A1 of Additional file 1. In reality, regulatory approval and with that a monetary gain, which is the core driver for our utility function, is achieved when a significant result is observed in phase III, acknowledging that there is a probability of *α* that it is a false positive decision. Thus, we keep the commonly used term “success” and *PsP* which should be regarded as probability of market access and not a probability of a correct decision.

### Considered program set-ups

We investigate the impact of using adjusted treatment effect estimates (i.e., ${\hat{\theta}}_2^{\lambda }$ or ${\hat{\theta}}_2^{\alpha_{CI}}$) for the go/no-go decision and/or for the calculation of the number of events for phase III on the drug development program characteristics and compare the results to those where the unadjusted (naïve) treatment effect estimate ${\hat{\theta}}_2^u$ was used. Therefore, we investigate different program set-ups $S\left({\hat{\theta}}_2^{s_1},{\hat{\theta}}_2^{s_2}\right)$ which are defined by the selection of the treatment effect estimate used for the decision rule (selection *s*_1_) and, in case of a go decision, by the choice of the treatment effect estimate used for the calculation of the number of events for phase III (selection *s*_2_).

Table 1 gives an overview of the considered program set-ups. We compare the “unadjusted” set-up $\left({\hat{\theta}}_2^u,{\hat{\theta}}_2^u\right)$, where ${\hat{\theta}}_2^u={\hat{\theta}}_2$ (i.e., *s*_1_, *s*_2_ = *u*), with two “multiplicatively adjusted” set-ups $S\left({\hat{\theta}}_2^{s_1},{\hat{\theta}}_2^{\lambda}\right)$ (*s*_1_ ∈ {*u*, *λ*}, *s*_2_ = *λ*), and two “additively adjusted” set-ups $S\left({\hat{\theta}}_2^{s_1},{\hat{\theta}}_2^{\alpha_{CI}}\right)$ (*s*_1_ ∈ {*u*, *α*_*CI*_}, *s*_2_ = *α*_*CI*_). Note that if *s*_1_ ≠ *u*, we define *s*_2_ = *s*_1_, which means that if an adjustment parameter is used for the decision rule, the same adjustment parameter is used for the calculation of the expected number of events for phase III (for reasons which will be given later).

**Table 1:** Overview of program set-ups $S\left({\hat{\theta}}_2^{s_1},{\hat{\theta}}_2^{s_2}\right)$

Program set-up $S\left({\hat{\theta}}_2^{s_1},{\hat{\theta}}_2^{s_2}\right)$	Adjustment of the estimate used for decision rule	Estimate used for decision rule	Adjustment of the estimate used for calculating the number of events for phase III	Estimate used for calculating the number of events for phase III
$S\left({\hat{\theta}}_2^u,{\hat{\theta}}_2^u\right)$ (unadjusted)	none (*s*_1_ = *u*)	${\hat{\theta}}_2^u$	none (*s*_2_ = *u*)	${\hat{\theta}}_2^u$
$S\left({\hat{\theta}}_2^u,{\hat{\theta}}_2^{\lambda}\right)$ (multiplicative)			multiplicative (*s*_2_ = *λ*)	${\hat{\theta}}_2^{\lambda }$
$S\left({\hat{\theta}}_2^u,{\hat{\theta}}_2^{\alpha_{CI}}\right)$ (additive)			additive (*s*_2_ = *α*_*CI*_)	${\hat{\theta}}_2^{\alpha_{CI}}$
$S\left({\hat{\theta}}_2^{\lambda },{\hat{\theta}}_2^{\lambda}\right)$ (multiplicative)	multiplicative (*s*_1_ = *λ*)	${\hat{\theta}}_2^{\lambda }$	multiplicative (*s*_2_ = *λ*)	${\hat{\theta}}_2^{\lambda }$
$S\left({\hat{\theta}}_2^{\alpha_{CI}},{\hat{\theta}}_2^{\alpha_{CI}}\right)$ (additive)	additive (*s*_1_ = *α*_*CI*_)	${\hat{\theta}}_2^{\alpha_{CI}}$	additive (*s*_2_ = *α*_*CI*_)	${\hat{\theta}}_2^{\alpha_{CI}}$

Program set-ups are defined by the estimate used for the go/no-go decision (selection *s*_1_: “go if ${\hat{\theta}}_2^{s_1}\ge \kappa$ ”) and by the calculation of the number of events for phase III (selection *s*_2_: ${D}_3\left({\hat{\theta}}_2^{s_2}\right),{s}_2\in \left\{\lambda, {\alpha}_{CI},u\right\}$, where ${\hat{\theta}}_2^{\lambda }={\hat{\theta}}_2\bullet \lambda$, ${\hat{\theta}}_2^{\alpha_{CI}}={\hat{\theta}}_2-{z}_{1-{\alpha}_{CI}}\bullet \sqrt{4/{d}_2}$, and ${\hat{\theta}}_2^u={\hat{\theta}}_2$ are the multiplicatively adjusted, additively adjusted, and unadjusted treatment effect estimates of phase II).

### Utility function

**Fig. 2:**
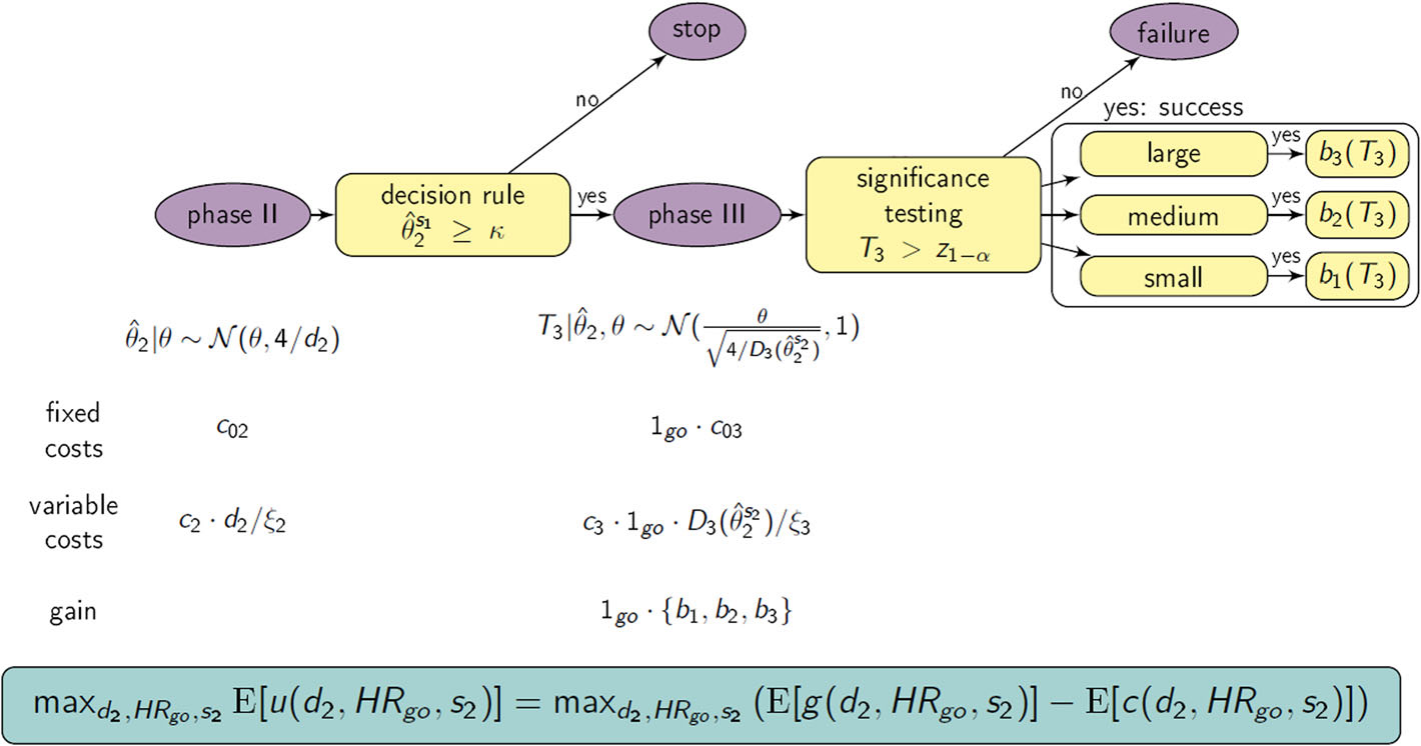
Graphical illustration of (adjusted) utility-based optimization. The treatment effect estimate of phase II may be adjusted for the decision rule (selection *s*_1_ ∈ {*u*, *s*_2_} and/or for the calculation of the number of events for phase III (selection *s*_2_ ∈ {*λ*, *α*_*CI*_, *u*}). The utility (including the costs and the gain) is optimized over the number of events for phase II *d*_2_, the threshold value for the decision rule *HR*_*go*_, and the adjustment parameter *s*_2_ = *λ* or *α*_*CI*_ (see Section 2.5 for details), *ξ*_*i*_ event rate in phase *i* = 2, 3, *b*_*j*_ = *b*_*j*_(*T*_3_) benefit categories *j* = 1, 2, 3

The aim is to optimize a phase II/III drug development program in terms of the adjustment parameters *λ* or *α*_*CI*_, the number of events in phase II *d*_2_, and the go/no-go decision threshold value *κ*. Therefore, we set up a utility function, which utilizes the difference between program costs and potential gains after successful market launch (compare Fig. 2 for a graphical illustration). For the costs, fixed (*c*_02_, *c*_03_) and variable per-patient (*c*_2_, *c*_3_) costs are included for the phase II and III trial, respectively. By dividing the number of events by the event rate *ξ*_*i*_, the total number of patients can be calculated for the respective phase *i* = 2, 3. Obviously, only in case of a go decision the costs of the phase III trial apply. In case of program success, a benefit *b* is obtained, and we assume that the level of benefit depends on the observed treatment effect in the phase III trial as suggested by a report of the German Institute for Quality and Efficiency in Health Care [29]. As proposed by them, three effect size categories (small, medium and large) are used, whereby each category is defined by a threshold value (1, 0.95, 0.85) for the upper boundary of the 95% confidence interval for the *HR* (for details on the derivation of these threshold values, the interested reader may be referred to the “Anhang A”of [29]). The corresponding amount of benefit is denoted by *b*_1_, *b*_2_ and *b*_3_, respectively. Based on this, costs *c*(*d*_2_, *κ*, *s*_2_) and gain *g*(*d*_2_, *κ*, *s*_2_) for a phase II/III program with program set-up $S\left({\hat{\theta}}_2^{s_1},{\hat{\theta}}_2^{s_2}\right)$ are given by

$$ c\left({d}_2,\kappa, {s}_2\right)={c}_{02}+\frac{d_2}{\xi_2}\cdot {c}_2+{1}_{\left\{{\hat{\theta}}_2^{s_1}\ge \kappa \right\}}\cdot \left({c}_{03}+\frac{D_3}{\xi_3}\cdot {c}_3\right) $$

$$ g\left({d}_2,\kappa, {s}_2\right)={1}_{\left\{{\hat{\theta}}_2^{s_1}\ge \kappa \right\}}\cdot \left({b}_1\cdot {1}_{\left\{{T}_3\in {I}_1\right\}}+{b}_2\cdot {1}_{\left\{{T}_3\in {I}_2\right\}}+{b}_3\cdot {1}_{\left\{{T}_3\in {I}_3\right\}}\right), $$

where ${I}_1=\left({z}_{1-\alpha },-\log (0.95)/\sqrt{4/{D}_3}+{z}_{1-\alpha}\right]$, ${I}_2=\left(-\log (0.95)/\sqrt{4/{D}_3}+{z}_{1-\alpha },-\log (0.85)/\sqrt{4/{D}_3}+{z}_{1-\alpha}\right]$ and ${I}_3=\left(-\log (0.85)/\sqrt{4/{D}_3}+{z}_{1-\alpha },\infty \right)$ are transformations of the effect size intervals to intervals on the test statistic scale of *T*_3_. Thus, the costs depend on the observed treatment effect in phase II and the gain depends on the observed treatment effect in phase II and III.

The utility is defined as the difference between costs and gain and expressed as a function of *d*_2_ and *κ* over which it is simultaneously optimized. In the adjusted program set-ups, the optimization is also over *λ* in the multiplicatively, and over *α*_*CI*_ in the additively adjusted set-ups, respectively. Thus, we define the utility for program set-up $S\left({\hat{\theta}}_2^{s_1},{\hat{\theta}}_2^{s_2}\right)$ by

$$ u\left({d}_2,\kappa, {s}_2\right)=g\left({d}_2,\kappa, {s}_2\right)-c\left({d}_2,\kappa, {s}_2\right), $$

where for the unadjusted program set-up $S\left({\hat{\theta}}_2^u,{\hat{\theta}}_2^u\right)$ *u*(*d*_2_, *κ*, *s*_2_) = *u*(*d*_2_, *κ*). To incorporate the development risk in terms of success probabilities, we consider the expected utility with respect to *θ*, ${\hat{\theta}}_2$ and *T*_3_ E[*u*(*d*_2_, *κ*, *s*_2_)] = E[*g*(*d*_2_, *κ*, *s*_2_)] − E[*c*(*d*_2_, *κ*, *s*_2_)], where the expected costs and gain with respect to *θ*, ${\hat{\theta}}_2$ and *T*_3_ are given by

$$ {\displaystyle \begin{array}{c}E\left[c\left({d}_2,\kappa, {s}_2\right)\right]={c}_{02}+{d}_2/{\xi}_2\cdot {c}_2+{c}_{03}\cdot {\int}_{-\infty}^{\infty }{\int}_{-\infty}^{\infty }{1}_{\left\{{\hat{\theta}}_2^{s_1}\ge \kappa \right\}}\cdot f\left({\hat{\theta}}_2|\theta \right)\cdot f\left(\theta \right)d{\hat{\theta}}_2 d\theta \\ {}+{c}_3/{\xi}_3\cdot {\int}_{-\infty}^{\infty }{\int}_{-\infty}^{\infty }{1}_{\left\{{\hat{\theta}}_2^{s_1}\ge \kappa \right\}}\cdot {D}_3\left({\hat{\theta}}_2^{s_2}\right)\cdot f\left({\hat{\theta}}_2|\theta \right)\cdot f\left(\theta \right)d{\hat{\theta}}_2 d\theta, \\ {}E\left[g\left({d}_2,\kappa, {s}_2\right)\right]=\sum \limits_{j=1}^3{b}_j{\int}_{-\infty}^{\infty }{\int}_{-\infty}^{\infty }{\int}_{-\infty}^{\infty }{1}_{\left\{{\hat{\theta}}_2^{s_1}\ge \kappa \right\}}\cdot {1}_{\left\{{T}_3\in {I}_j\right\}}\cdot f\left({t}_3|{\hat{\theta}}_2,\theta \right)\cdot f\left({\hat{\theta}}_2|\theta \right)\cdot f\left(\theta \right)d{t}_3d{\hat{\theta}}_2 d\theta .\end{array}} $$

The aim is to find a design *δ* = (*d*_2_, *κ*, *s*_2_) that maximizes the expected utility E[*u*(*d*_2_, *κ*, *s*_2_)] for programs with program set-up $S\left({\hat{\theta}}_2^{s_1},{\hat{\theta}}_2^{s_2}\right).$ The optimization is carried out over *d*_2_, *κ*, and *λ* in the multiplicatively or *α*_*CI*_ in the additively adjusted set-ups, respectively. The optimal design *δ*^∗^ for each program set-up $S\left({\hat{\theta}}_2^{s_1},{\hat{\theta}}_2^{s_2}\right)$ is defined to be the design for which the expected utility is maximized, that is, $\mathrm{E}\left[u\left({\delta}^{\ast}\right)\right]=\underset{\delta \in D}{\max}\mathrm{E}\left[u\left(\delta \right)\right]$, where *D* = {*δ* = (*d*_2_, *κ*, *s*_2_)} is the optimization set.

The optimization is solved by using numerical integration procedures written in the programming language R [30]. In order to facilitate the application of the approach, an user friendly R Shiny App (bias) and an R package (drugdevelopR including the R function optimal_bias) are provided open-source (both assessable via [1]).

### Illustration of the framework by application to oncology trial example and practical extensions

In this paper, the parameters in the oncology trial example are chosen as in Kirchner et al. [27] to allow comparison of results. It should be noted that the example is primarily given to illustrate the framework and the chosen parameters should not be taken as face values. We tried to elicit the design parameters as realistic as possible to mimic an oncology drug development program by means of information from relevant literature and consultation with experts from the pharmaceutical industry in the field of oncology. However, it should be noted, that these parameters must be chosen carefully and specifically for each drug development scenario at hand.

The event rates for phase II and III are set to *ξ*_*i*_ = 0.7 for *i* = 2, 3. Therefore, the total sample size can be calculated by *d*_*i*_/0.7, *i* = 2, 3. In practice, estimates on the event rates could be obtained by taking recruitment rates and duration as well as drop-out rates and treatment group specific hazards into account. However, using those parameters often leads to event rates around *ξ*_*i*_ = 0.7 as it is a compromise between data maturity and avoidance of long follow-up times if drop-out rates are higher than expected. If *ξ*_*i*_ < 0.5 the median event time might not be observed while if *ξ*_*i*_ is too high, the planned number of events might not be reached at all with substantial drop-out rates.

For phase III oncology trials, per-patient costs between 75,000 and 125,000 US *$* are reported [31]. Therefore, per-patient costs for phase III of *$*10^5^ are considered and *c*_3_ is set to 1 (in *$*10^5^). Furthermore, the per-patient costs for phase II are set to *c*_2_ = 0.75 (in *$*10^5^). Due to, for example, additional biomarker measurements made in phase III, or because regulatory agencies may require more extensive data collection in phase III [32], higher per-patient costs in phase III compared with phase II are reasonable. In this example, the fixed costs for phase II and III are set to *c*_02_ = 100 and *c*_03_ = 150 (in *$*10^5^), respectively. To investigate different scenarios, the benefit parameters *b*_1_, *b*_2_ and *b*_3_ are chosen to embody a low (*b*_1_, *b*_2_, *b*_3_) 1 : (1000, 2000, 3000), 2 : (1000, 2000, 4000), 3 : (1000, 3000, 4000) and a medium to large (*b*_1_, *b*_2_, *b*_3_) = 4 : (1000, 3000, 5000), 5 : (1000, 4000, 5000), 6 : (1000, 3000, 6000), 7 : (1000, 4000, 6000) over-all benefit (in *$*10^5^), where we assume a 5-year income period and profit margin of 0.2. Thus, seven different benefit scenarios (*bs* 1–7) will be considered. A mixture distribution consisting of the weighted sum of two normal distributions

$$ \theta \sim w\cdotp N\left(-\mathit{\log}(0.69),\left(4/210\right)\right)+\left(1-w\right)\cdotp N\left(-\mathit{\log}(0.88),\left(4/420\right)\right), $$

as proposed by Götte et al. [26] can be used to model the true treatment effect. The two normal distributions each depict a distribution for *θ*, whereby the means represent values of the assumed true treatment effect and the denominators of the associated variances can be viewed as “amount of certainty” about the treatment effect size in terms of numbers of events. The parameters of the distributions (i.e., means and variances) are elected such that a realistic range for the *HR* is covered (compare Fig. A2 in Additional file 1 and/or investigate the prior distribution with the help of our R shiny App prior [33]). The mean of the first of the two normal distributions characterizes a strong, the second one a moderate to low treatment effect, so that by ranging *w* from, e.g., 0.3 to 0.9 we can mirror pessimistic to more optimistic opinions about the true treatment effect. In practice, the choice of *w* can be guided by formal expert elicitation methods. Dallow et al. [34] presented an overview of such methods including elicitation of Gaussian mixture distributions. Note that the approach is general and allows for implementation of any alternative prior distribution. Again, elicitation methods (compare also, e.g., [35]) are a useful tool that may help (a group of) experts to quantify their opinions about the treatment effect as a probability distribution. Various software packages enable their practical application (compare, e.g., [36]).

In our framework it is also possible to account for, e.g., different population structures in phase II and phase III (due to different countries, centers, in-/exclusion criteria, …) by assuming different distributions for the assumed true treatment effect in phase II and III (i.e., *θ*_2_ ≁ *θ*_3_), so that ${\hat{\theta}}_2\mid {\theta}_2\sim N\left({\theta}_2,4/{d}_2\right)$ and ${T}_3\mid {\hat{\theta}}_2,{\theta}_2,{\theta}_3\sim N\left({\theta}_3/\sqrt{4/{D}_3},1\right)$. For ease of interpretation, all formulas and results presented in the main part are for the special case, where the true treatment effect is modelled by the same distribution for phase II and III (e.g., *θ*~*θ*_2_~*θ*_3_), and a brief investigation of this aspect can be found in Section A2 of Additional file 1.

In this example, we chose a wide range for *κ* (and *d*_2_, as well as *λ* or *α*_*CI*_, respectively) such that the optimization is not influenced by that choice. Therefore, the optimization set is D ={*δ* = (*d*_2_, *κ*, *s*_2_), *d*_2_ ∈ {50, 52, …, 350}, *κ* ∈ {− log(0.9), − log(0.89), …, − log(0.7)}, *s*_2_ = *λ* ∈ {0.2, 0.225, …, 1} or *s*_2_ = *α*_*CI*_ ∈ {0.025, 0.075, …, 0.5}}. However, the lower bound of the decision rule set for *κ* can also be seen as representing a predefined clinically relevant effect size: phase III trials are then only conducted if the treatment effect observed in phase II is at least of that size. In Section A3 of Additional file 1, we present results of the procedure, where we chose min(*κ*) =  − log(0.8). Furthermore, it might be interesting to see how the optimal program design is influenced by the sponsor’s real life budget constraint. Therefore, we also consider optimizing the drug development program with a constraint *K* on the expected costs of the program, i.e., E[*c*(*d*_2_, *κ*, *s*_2_)] ≤ *K* (see Section A4 of Additional file 1 for more details). In pharmaceutical industry there are often discussions about skipping the phase II trial. For example, if competitors have already approved a drug with a similar mode of action one might see no need for further learning about the drug and go directly to a confirmatory trial. Our framework allows to systematically assess this aspect by setting *d*_2_ = 0, *c*_02_ = *c*_2_ = 0 and *p*_*go*_ = 1 (see Section A5 of Additional file 1 for more details). In addition, different definitions of the cost and benefit functions are possible. As mentioned above, the choice of three effect size categories (and therefore the benefit function) is based on a report of the German Institute for Quality and Efficiency in Health Care [29]. However, the presented framework could also be applied to an alternative set-up as, for example, the one proposed by Ding et al. [32]. Here, a proportional relationship between benefit and effect size is considered. In Section A6 of Additional file 1 we investigate this possibility in more detail.

## Results

This section is organized as follows. It starts with general observations across all program set-ups $S\left({\hat{\theta}}_2^{s_1},{\hat{\theta}}_2^{s_2}\right)$. Then, we compare multiplicative $S\left({\hat{\theta}}_2^{s_1},{\hat{\theta}}_2^{\lambda}\right)$ vs. additive $S\left({\hat{\theta}}_2^{s_1},{\hat{\theta}}_2^{\alpha_{CI}}\right)$ vs. no adjustment $S\left({\hat{\theta}}_2^u,{\hat{\theta}}_2^u\right)$, where *s*_1_ = *u* or *s*_1_ = *s*_2_. The impact of adjusting the go/no go decision making, i.e., the differences between both multiplicative ($S\left({\hat{\theta}}_2^u,{\hat{\theta}}_2^{\lambda}\right)$ vs. $S\left({\hat{\theta}}_2^{\lambda },{\hat{\theta}}_2^{\lambda}\right)$) and both additive adjustment methods ($S\left({\hat{\theta}}_2^u,{\hat{\theta}}_2^{\alpha_{CI}}\right)$ vs. $S\left({\hat{\theta}}_2^{\alpha_{CI}},{\hat{\theta}}_2^{\alpha_{CI}}\right)$) are also presented. A discussion of the results is given in the next section.

**Fig. 3:**
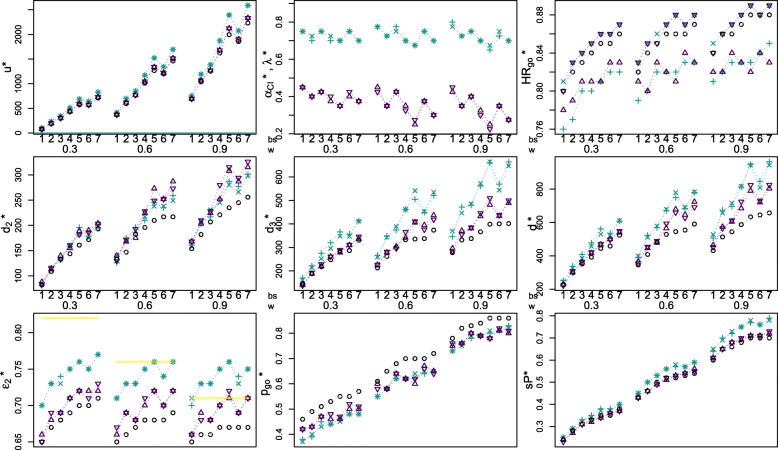
Optimization results. Maximal expected utility *u*^∗^, corresponding optimal design parameters $$ {\delta}^{\ast }=\left({d}_2^{\ast },{HR}_{go}^{\ast}\right) $$, $$ {\delta}^{\ast }=\left({d}_2^{\ast },{HR}_{go}^{\ast },{\lambda}^{\ast}\right) $$ or $$ {\delta}^{\ast }=\left({d}_2^{\ast },{HR}_{go}^{\ast },{\alpha}_{CI}^{\ast}\right) $$, expected probability to go to phase III $$ {p}_{go}^{\ast } $$, expected probability of a successful program *sP*^∗^, expected estimate used for sample size calculation $$ {\varepsilon}_2^{\ast } $$, expected number of events in phase III when going to phase III $$ {d}_3^{\ast } $$ and expected total number of events of program *d*^∗^ in the optimal design, for *c*_2_ = 0.75, *c*_3_ = 1, *c*_02_ = 100, *c*_03_ = 150 in $ 10^5^, *ξ*_2_ = *ξ*_3_ = 0.7, 1 − *β* = 0.9, *α* = 0.025 (one sided), for program set-ups $$ S\left({\hat{\theta}}_2^{s_1},{\hat{\theta}}_2^{s_2}\right) $$, *s*_1_, *s*_2_ = *λ*, *α*_*CI*_ or *u* (that is $$ S\left({\hat{\theta}}_2^u,{\hat{\theta}}_2^u\right) $$: black circle; $$ S\left({\hat{\theta}}_2^u,{\hat{\theta}}_2^{\lambda}\right) $$, $$ S\left({\hat{\theta}}_2^{\lambda },{\hat{\theta}}_2^{\lambda}\right) $$: green cross; $$ S\left({\hat{\theta}}_2^u,{\hat{\theta}}_2^{\alpha_{CI}}\right) $$, $$ S\left({\hat{\theta}}_2^{\alpha_{CI}},{\hat{\theta}}_2^{\alpha_{CI}}\right) $$: violet triangle), benefit scenarios *bs* 1–7, and weights for the prior distribution *w* = 0.3, 0.6, 0.9, where the yellow line indicates $$ \exp \left(-\mathrm{E}\left[{\hat{\theta}}_2\right]\right) $$. Note that the symbols used to show the program characteristics of both multiplicatively and additively adjusted program set-ups, i.e., green crosses and violet triangles, appear as stars when plotted on top of each other

The optimization results are presented in Table 2 (naïve setting, multiplicative adjustment), Table 3 (additive adjustment) and Figure 3, which show the optimal design parameters ${\delta}^{\ast }=\left({d}_2^{\ast },{\kappa}^{\ast },{s}_2^{\ast}\right)$:

- optimal total number of events for phase II ${d}_2^{\ast }$ (given by the optimal value of *d*_2_ ∈ *D*),
- optimal go/no-go decision rule threshold value ${HR}_{go}^{\ast }$ (given by the optimal value of *κ* ∈ *D* in “HR-scale”, i.e., ${HR}_{go}^{\ast }=\exp \left(-{\kappa}^{\ast}\right)$) and
- optimal adjustment parameter ${s}_2^{\ast}\in \left\{{\lambda}^{\ast },{a}_{CI}^{\ast}\right\}$ (given by the optimal value of *s*_2_ ∈ *D*) for the multiplicative and additive adjustment method, respectively,

with corresponding program characteristics for the optimal design:

- maximal expected utility *u*^∗^ = *E*[*u*(*δ*^∗^)],
- expected number of events for phase III ${d}_3^{\ast }={d}_3\left({\hat{\theta}}_2^{s_1},{\hat{\theta}}_2^{s_2^{\ast }}\right)$, where we chose a desired power of 1 − *β* = 0.9 and a one-sided significance level *α* = 0.025,
- total number of expected events in the program ${d}^{\ast }={d}_3^{\ast }+{d}_2^{\ast }$,
- expected probability to go to phase III ${p}_{go}^{\ast }={p}_{go}\left({\hat{\theta}}_2^{s_1}\right)$,
- expected probability of a successful program ${sP}^{\ast }= PsP\left({\hat{\theta}}_2^{s_1},{\hat{\theta}}_2^{s_2^{\ast }}\right)$ and
- expected estimate of phase II used for sample size calculation ${\varepsilon}_2^{\ast }=\exp \left(-{e}_2\left({\hat{\theta}}_2^{s_1},{\hat{\theta}}_2^{s_2^{\ast }}\right)\right)$ in “HR-scale”,

for program set-ups $S\left({\hat{\theta}}_2^{s_1},{\hat{\theta}}_2^{s_2}\right)$, where *s*_1_ = *u* or ${s}_1={s}_2^{\ast}\in \left\{{\lambda}^{\ast },{a}_{CI}^{\ast}\right\}$, benefit scenarios (*bs* 1-7) and weights for the prior distribution of the true underlying effect (*w* = 0.3, 0.6, 0.9), where $E\left[{\hat{\theta}}_2\right]={\int}_{-\infty}^{\infty }{\int}_{-\infty}^{\infty }{\hat{\theta}}_2\cdot f\left({\hat{\theta}}_2|\theta \right)\cdot f\left(\theta \right)d{\hat{\theta}}_2 d\theta$*.*

**Table 2:** Optimal design parameters for unadjusted and multiplicatively adjusted program set-ups

	Unadjusted								Multiplicatively adjusted																	
	Program set-up $S\left({\hat{\theta}}_2^u,{\hat{\theta}}_2^u\right)$								Program set-up $S\left({\hat{\theta}}_2^u,{\hat{\theta}}_2^{\lambda}\right)$									Program set-up $S\left({\hat{\theta}}_2^{\lambda },{\hat{\theta}}_2^{\lambda}\right)$								
*bs*	${HR}_{go}^{\ast }$	${d}_2^{\ast }$	${\varepsilon}_2^{\ast }$	${d}_3^{\ast }$	*d*^∗^	${p}_{go}^{\ast }$	*sP*^∗^	*u*^∗^	*λ*^∗^	${HR}_{go}^{\ast }$	${d}_2^{\ast }$	${\varepsilon}_2^{\ast }$	${d}_3^{\ast }$	*d*^∗^	${p}_{go}^{\ast }$	*sP*^∗^	*u*^∗^	*λ*^∗^	${HR}_{go}^{\ast }$	${d}_2^{\ast }$	${\varepsilon}_2^{\ast }$	${d}_3^{\ast }$	*d*^∗^	${p}_{go}^{\ast }$	*sP*^∗^	*u*^∗^
***w = .*3**, i.e., $\mathbf{\exp}\left(-\mathbf{E}\left[{\hat{\boldsymbol{\theta}}}_{\mathbf{2}}\right]\right)=.\mathbf{82}$																										
1	.80	82	.65	146	228	.46	.24	76	.750	.76	81	.70	170	251	.38	.25	99	.750	.81	84	.70	161	245	.37	.25	100
2	.82	109	.67	189	298	.49	.28	188	.700	.77	116	.73	222	338	.39	.29	235	.725	.83	112	.73	214	326	.40	.29	235
3	.83	133	.68	218	351	.51	.31	299	.750	.80	133	.74	275	408	.45	.33	343	.750	.84	133	.73	252	385	.43	.32	343
4	.84	144	.69	248	392	.53	.33	432	.700	.80	158	.75	320	478	.44	.35	509	.725	.85	161	.75	296	457	.44	.34	508
5	.85	161	.70	284	445	.55	.35	569	.700	.81	196	.76	366	562	.46	.38	690	.700	.86	182	.76	348	530	.45	.37	690
6	.85	172	.70	287	459	.55	.35	567	.750	.82	179	.75	357	536	.48	.38	640	.750	.86	175	.75	347	522	.48	.37	640
7	.86	193	.71	331	524	.57	.38	712	.700	.82	196	.77	413	609	.48	.40	828	.700	.87	200	.77	412	612	.48	.40	828
***w = .*6**, i.e., $\mathbf{\exp}\left(-\mathbf{E}\left[{\hat{\boldsymbol{\theta}}}_{\mathbf{2}}\right]\right)=.\mathbf{76}$																										
1	.82	133	.65	213	346	.61	.43	370	.775	.79	126	.71	265	391	.55	.45	412	.775	.83	140	.71	259	399	.55	.45	411
2	.84	147	.66	262	409	.65	.46	598	.725	.80	175	.73	348	523	.58	.50	696	.725	.85	168	.73	343	511	.58	.50	696
3	.85	182	.67	299	481	.68	.50	764	.775	.82	196	.73	374	570	.62	.53	849	.750	.86	189	.73	390	579	.62	.53	849
4	.86	196	.68	333	529	.70	.52	1012	.700	.82	210	.75	462	672	.62	.56	1172	.700	.87	217	.75	462	679	.62	.56	1172
5	.86	210	.68	336	546	.70	.52	1267	.675	.82	245	.76	505	750	.62	.57	1523	.675	.88	238	.76	542	780	.64	.58	1521
6	.86	217	.68	338	555	.70	.53	1200	.750	.83	235	.74	450	685	.64	.57	1342	.750	.87	238	.74	453	691	.65	.57	1343
7	.87	217	.69	374	591	.72	.54	1460	.700	.83	259	.76	521	780	.65	.59	1693	.700	.88	249	.76	535	784	.65	.59	1693
***w = .*9**, i.e., $\mathbf{\exp}\left(-\mathbf{E}\left[{\hat{\boldsymbol{\theta}}}_{\mathbf{2}}\right]\right)=.\mathbf{71}$																										
1	.84	154	.65	278	432	.78	.60	693	.800	.81	161	.70	346	507	.73	.64	753	.775	.85	158	.71	370	528	.73	.65	753
2	.86	182	.66	332	514	.82	.65	1039	.725	.82	203	.73	472	675	.76	.70	1193	.725	.86	210	.73	447	657	.75	.69	1193
3	.86	207	.66	338	545	.83	.66	1255	.750	.83	217	.73	480	697	.78	.72	1384	.750	.87	231	.73	486	717	.79	.73	1384
4	.87	221	.67	367	588	.84	.68	1623	.700	.83	252	.75	562	814	.79	.75	1871	.700	.88	245	.75	573	818	.79	.75	1871
5	.88	235	.67	399	634	.86	.70	1996	.650	.83	287	.76	661	948	.80	.77	2394	.675	.89	280	.76	665	945	.81	.78	2394
6	.88	245	.67	401	646	.86	.70	1855	.725	.84	277	.74	570	847	.81	.76	2072	.750	.88	266	.73	544	810	.81	.76	2072
7	.88	256	.67	402	658	.86	.70	2233	.700	.85	301	.75	664	965	.83	.79	2589	.700	.89	298	.75	647	945	.82	.78	2590

Optimal design parameters *λ*^∗^, $$ {d}_2^{\ast } $$ and $$ {HR}_{go}^{\ast } $$, corresponding value of maximal expected utility *u*^∗^, expected estimate used for sample size calculation $$ {\varepsilon}_2^{\ast } $$, expected number of events in phase III when going to phase III $$ {d}_3^{\ast } $$, expected total number of events of program *d*^∗^, expected probability to go to phase III $$ {p}_{go}^{\ast } $$, and expected probability of a successful program *sP*^∗^ for the optimal design, for *c*_2_ = 0.75, *c*_3_ = 1, *c*_02_ = 100, *c*_03_ = 150 in $ 10^5^, *ξ*_2_ = *ξ*_3_ = 0.7, 1 − *β* = 0.9, *α* = 0.025 (one sided), benefit scenarios *bs* 1–7, weights for the prior distribution *w* = 0.3, 0.6, 0.9, for the unadjusted program set-up $$ S\left({\hat{\theta}}_2^u,{\hat{\theta}}_2^u\right) $$ and multiplicatively adjusted program set-ups $$ S\left({\hat{\theta}}_2^{s_1},{\hat{\theta}}_2^{\lambda}\right) $$, respectively

**Table 3:** Optimal design parameters for additively adjusted program set-ups

Additively adjusted																		
Program set-up $S\left({\hat{\theta}}_2^u,{\hat{\theta}}_2^{\alpha_{CI}}\right)$										Program set-up $S\left({\hat{\theta}}_2^{\alpha_{CI}},{\hat{\theta}}_2^{\alpha_{CI}}\right)$								
*bs*	*α*_*CI*_^∗^	${HR}_{go}^{\ast }$	${d}_2^{\ast }$	${\varepsilon}_2^{\ast }$	${d}_3^{\ast }$	*d*^∗^	${p}_{go}^{\ast }$	*sP*^∗^	*u*^∗^	*α*_*CI*_^∗^	${HR}_{go}^{\ast }$	${d}_2^{\ast }$	${\varepsilon}_2^{\ast }$	${d}_3^{\ast }$	*d*^∗^	${p}_{go}^{\ast }$	*sP*^∗^	*u*^∗^
***w = .*3**, i.e., $\mathbf{\exp}\left(-\mathbf{E}\left[{\hat{\boldsymbol{\theta}}}_{\mathbf{2}}\right]\right)=.\mathbf{82}$																		
1	.450	.78	88	.66	140	228	.42	.24	78	.450	.80	84	.65	138	222	.42	.23	78
2	.400	.79	113	.68	188	301	.43	.27	194	.400	.83	116	.69	192	308	.43	.28	194
3	.425	.81	140	.69	220	360	.47	.31	302	.425	.84	133	.69	229	362	.47	.31	302
4	.375	.81	155	.71	261	416	.46	.32	442	.400	.85	161	.71	261	422	.48	.33	442
5	.350	.81	189	.72	278	467	.46	.34	593	.350	.86	182	.72	289	471	.47	.34	593
6	.400	.83	190	.72	310	500	.50	.36	573	.425	.86	186	.71	311	497	.52	.36	573
7	.375	.83	204	.72	336	540	.50	.37	729	.375	.87	203	.73	346	549	.51	.37	729
***w = .*6**, i.e., $\mathbf{\exp}\left(-\mathbf{E}\left[{\hat{\boldsymbol{\theta}}}_{\mathbf{2}}\right]\right)=.\mathbf{76}$																		
1	.450	.81	140	.66	226	366	.60	.43	372	.425	.83	130	.67	226	356	.58	.43	372
2	.350	.80	168	.69	278	446	.58	.46	614	.350	.85	172	.69	282	454	.58	.46	614
3	.425	.83	175	.68	304	479	.64	.49	772	.425	.85	193	.68	296	489	.64	.49	772
4	.350	.82	224	.70	341	565	.62	.51	1045	.325	.87	228	.71	366	594	.62	.52	1045
5	.250	.81	273	.72	406	679	.60	.53	1338	.275	.88	249	.72	411	660	.62	.53	1338
6	.375	.84	252	.70	395	647	.67	.54	1221	.375	.87	252	.70	376	628	.66	.54	1222
7	.300	.83	287	.72	437	724	.65	.56	1515	.300	.88	273	.72	419	692	.64	.55	1515
***w = .*9**, i.e., $\mathbf{\exp}\left(-\mathbf{E}\left[{\hat{\boldsymbol{\theta}}}_{\mathbf{2}}\right]\right)=.\mathbf{71}$																		
1	.425	.82	168	.66	296	464	.75	.61	695	.450	.84	168	.66	284	452	.76	.61	695
2	.350	.82	203	.69	371	574	.76	.65	1068	.350	.86	210	.68	355	565	.76	.65	1068
3	.400	.84	224	.68	381	605	.80	.68	1272	.400	.87	228	.68	385	613	.80	.68	1272
4	.325	.83	252	.70	433	685	.79	.69	1681	.300	.88	280	.70	446	726	.79	.70	1682
5	.250	.82	308	.71	482	790	.78	.71	2122	.225	.89	315	.72	510	825	.78	.71	2122
6	.350	.84	287	.69	434	721	.81	.71	1898	.350	.88	294	.69	438	732	.82	.71	1898
7	.275	.83	315	.71	489	804	.80	.72	2333	.275	.89	326	.71	500	826	.81	.73	2334

Optimal design parameters *α*_*CI*_^*∗*^, $$ {d}_2^{\ast } $$ and $$ {HR}_{go}^{\ast } $$, corresponding value of expected utility *u*^*∗*^, expected estimate used for sample size calculation $$ {\varepsilon}_2^{\ast } $$, expected number of events in phase III when going to phase III $$ {d}_3^{\ast } $$, expected total number of events of program *d*^*∗*^, expected probability to go to phase III $$ {p}_{go}^{\ast } $$, and expected probability of a successful program *sP*^*∗*^ for the optimal design, for *c*_2_ = 0.75*,c*_3_ = 1, *c*_02_ = 100*,c*_03_ = 150 in $ 10^5^, *ξ*_2_ *= ξ*_3_ = 0.7, 1 *− β =* 0.9, α = 0.025 (one sided), benefit scenarios *bs* 1–7, weights for the prior distribution *w* = 0.3, 0.6, 0.9 for the additively adjusted program set-ups $$ S\left({\hat{\theta}}_2^{s_1},{\hat{\theta}}_2^{\alpha_{CI}}\right) $$

Overall, larger assumed benefits (i.e., larger values for (*b*_1_, *b*_2_, *b*_3_)) lead to more liberal optimal decision rules (i.e., larger values for ${HR}_{go}^{\ast }$) and higher investment in phase II (i.e., larger number of events for phase II ${d}_2^{\ast }$). This leads to a larger investment (in phase III), i.e., a higher expected probability to go to phase III ${p}_{go}^{\ast }$ and a larger expected number of events in phase III ${d}_3^{\ast }$, respectively. This results in a larger expected probability of a successful program *sP*^∗^ and thus in a larger maximal expected utility *u*^∗^.

In the multiplicatively adjusted program set-ups $S\left({\hat{\theta}}_2^{s_1},{\hat{\theta}}_2^{\lambda}\right)$, the maximal expected utility is always higher than the maximal expected utility in the additively adjusted program set-ups $S\left({\hat{\theta}}_2^{s_1},{\hat{\theta}}_2^{\alpha_{CI}}\right)$, which in turn is always higher than the maximal expected utility in the unadjusted program set-up $S\left({\hat{\theta}}_2^u,{\hat{\theta}}_2^u\right)$. It stands out that the investment in terms of numbers of events (i.e., ${d}_2^{\ast },{d}_3^{\ast },{d}^{\ast }$) tends to be higher in the adjusted program set-ups compared to the unadjusted program set-up, especially for scenarios with higher benefits and more optimistic prior. The expected probability to go to phase III ${p}_{go}^{\ast }$ is notably lower in the adjusted program set-ups compared to the unadjusted program set-up, whereas the expected probability of a successful program *sP*^∗^ is higher.

Dividing the optimal number of events in phase II by the expected number of events in phase III (i.e., ${d}_2^{\ast }$ / ${d}_3^{\ast }$), leads to values of 0.55–0.64, 0.55–0.64, 0.58–0.67, 0.43–0.54 and 0.42–0.54 in program set-up $S\left({\hat{\theta}}_2^u,{\hat{\theta}}_2^u\right)$, $S\left({\hat{\theta}}_2^u,{\hat{\theta}}_2^{\alpha_{CI}}\right)$, $S\left({\hat{\theta}}_2^{\alpha_{CI}},{\hat{\theta}}_2^{\alpha_{CI}}\right)$, $S\left({\hat{\theta}}_2^u,{\hat{\theta}}_2^{\lambda}\right)$ and $S\left({\hat{\theta}}_2^{\lambda },{\hat{\theta}}_2^{\lambda}\right)$, respectively. Furthermore, it can be observed that the treatment effect estimate of phase II used for sample size calculation in the optimal design is overestimated in the unadjusted setting (${\varepsilon}_2^{\ast }<\exp \left(-\mathrm{E}\left[{\hat{\theta}}_2\right]\right)$ as indicated by the black circles and yellow line in Figure 3). This overestimation is lower in the adjusted settings and can even result in an underestimation (compare multiplicative settings for *w* = 0.9).

The operating characteristics for the optimal designs (e.g., *u*^∗^, *sP*^∗^) compared between the two multiplicatively and the two additively adjusted program set-ups do not vary (much) for each benefit scenario *bs* and choice of weight for the prior distribution *w*, respectively. However, there are differences in the optimal choice of the threshold value for the decision rule ${HR}_{go}^{\ast }$: in the program set-ups with adjusted phase II treatment effect estimate used for decision making ($S\left({\hat{\theta}}_2^{\lambda },{\hat{\theta}}_2^{\lambda}\right)$ and $S\left({\hat{\theta}}_2^{\alpha_{CI}},{\hat{\theta}}_2^{\alpha_{CI}}\right)$), ${HR}_{go}^{\ast }$ is always larger (by 0.04 to 0.06 and by 0.01 to 0.07, respectively) than in the program set-ups with unadjusted treatment effect used for decision making ($S\left({\hat{\theta}}_2^u,{\hat{\theta}}_2^{\lambda}\right)$ and $S\left({\hat{\theta}}_2^u,{\hat{\theta}}_2^{\alpha_{CI}}\right)$).

## Discussion

To find optimal drug development designs, the costs of the program (fixed/variable costs for phase II/III), the assumed benefit, and the development risk (i.e., the expected probability of a successful program) are taken into account. By maximizing the expected utility with respect to the design parameters (adjustment parameter, number of events for phase II and threshold value for the go/no-go decision rule), optimal phase II/III drug development program designs can be found. Therefore, it enables quantitative reasoning for the design (i.e., the optimal “amount of adjustment”, sample size and decision rule) for specific drug development programs at hand.

We investigated two adjustment methods (additive and multiplicative adjustment), several benefit scenarios (e.g., low, medium, large overall benefit), different distributions for the true treatment effect (with the same and different distributions in phase II and III), scenarios with a real life budget constraint, scenarios with a predefined clinically relevant effect, and scenarios where phase II could be skipped, hence presented a method for the implementation of a variety of possible oncology drug development program scenarios, and an opportunity for assessing associated changes of the optimal design parameters. Of course, the implementation of alternative (e.g., proportional relationship between benefit and effect size) or more complex planning situations and broader application to other research areas are possible by choosing relevant (e.g., cost and benefit) parameters appropriately [37–39]. As the framework has been shown to be very flexible, frequent scenarios in oncology drug development are adequately mapped with our approach. However, certain situations may be simplified. For example, in our framework the development program consists entirely of just one phase II trial and one phase III trial, which is, however, not unusual in oncology. For situations that two or more phase III trials are performed, the framework of optimal planning of development programs was presented in a recent article by Preussler et al. [40]. Furthermore, we assumed the phase II trial to be two-armed. In the field of oncology dose investigations are often performed before and not as a part of phase II. However, in other indications dose-finding is performed in phase II. Methods for optimizing phase II/III programs with multi-armed phase II/III studies are presented in Preussler et al. [41]. Futility investigations in the phase III trial and/or considering a “seamless design” for the final analysis may be a worthwhile option, and it will be a topic of future research to investigate their impact on the optimal design. We assumed that the endpoint used in phase II and phase III is the same. We are currently exploring the situation that a surrogate (like progression-free or disease-free survival) is captured in phase II and overall survival is the primary endpoint in phase III. Another important point is that time-effects are not considered in this article. The program is unaccounted for the duration of development which is amongst others discussed in Preussler et al. [41]. That work presents in detail how to incorporate the impact of trial duration into the framework (compare Supplementary Material A2 [41]). However, when trying to incorporate “time” into the utility function, many aspects have to be considered. For example, one could take into account the “life cycle” of a drug as proposed by Patel & Ankolekar [42] who describe a typical life cycle by an early growth phase followed by a plateau, after which the sales decline as the patent expires. Furthermore, if there are several competitors investigating a similar drug then the company, who is the first to bring the drug to the market, usually gets the higher market share, i.e., higher gain. However, including these aspects requires competitor information and assumptions about their unknown future observed treatment effects. Any such assumptions are usually associated with very high uncertainty. Instead of trying to include too many (unknown) aspects into the utility function a rather simplified approach, as presented here, is advisable. If after observing phase II data further information about the potential of the drug, dose, target population or (time-dependent) benefits are available the probability of success (compare [43]) and the utility function could be updated to support go/no-go decisions as well as the design of the phase III trial.

In general, our results show that the adjusted program set-ups are superior to the unadjusted program set-up with respect to the maximal expected utility. This is associated with higher investments in terms of number of events and lower expected probabilities to go to phase III in the adjusted program set-ups compared to the unadjusted approach. Thus, in the adjusted program set-ups it is less often decided to go to phase III, but in case of a go decision, the investment in terms of sample size is higher. These aspects are particularly true for the multiplicatively adjusted program set-ups, which have also higher expected probabilities of a successful program compared to the additively adjusted and unadjusted program set-ups. Simply said, the money is spent more wisely when adjustment methods are used.

Values for the adjustment parameters that do not lead to an adjustment (i.e., *α*_*CI*_ = 0.5 and *λ* = 1 in the additively and multiplicatively adjusted program set-ups, respectively) were included but never selected in the optimization. Thus, the results suggest that adjustment should always be considered, which is in line with Chuang-Stein and Kirby [14]. Furthermore, we see that in the unadjusted case there is an overestimation of the treatment effect after phase II, which is mitigated by the adjustments. In the multiplicative setting it is even shown that an overcorrection and thus an even larger investment in terms of sample size can be worthwhile with respect to the expected utility. Note that the focus is on maximal expected utility and the expected estimate of phase II is only a supporting variable, i.e., obtaining a “perfectly” unbiased estimator is not the goal in this application. With regard to the optimal number of events in phase II compared to phase III (${d}_2^{\ast }$ / ${d}_3^{\ast }$), it can be seen that with the framework in the unadjusted and additive case one ends up in the “desirable” (according to De Martini [4, 25]) range of 2/3 and also in the multiplicative case with lower ${d}_2^{\ast }$ / ${d}_3^{\ast }$, one still exceeds the often used 1/4. However, it should be noted that the total optimal sample size is highest for the multiplicative case.

Both multiplicatively adjusted (i.e., $S\left({\hat{\theta}}_2^{s_1},{\hat{\theta}}_2^{\lambda}\right)$) and additively adjusted (i.e., $S\left({\hat{\theta}}_2^{s_1},{\hat{\theta}}_2^{\alpha_{CI}}\right)$) program set-ups do not differ in their maximal expected utility, whereas the program set-ups with adjusted estimate used for decision making (i.e., $S\left({\hat{\theta}}_2^{\lambda },{\hat{\theta}}_2^{\lambda}\right)$ and $S\left({\hat{\theta}}_2^{\alpha_{CI}},{\hat{\theta}}_2^{\alpha_{CI}}\right)$) have larger optimal threshold values for the decision rule than program set-ups where only the estimate used for calculating the expected number of events for phase III is adjusted (i.e., $S\left({\hat{\theta}}_2^u,{\hat{\theta}}_2^{\lambda}\right)$ and $S\left({\hat{\theta}}_2^u,{\hat{\theta}}_2^{\alpha_{CI}}\right)$). Considering only these two aspects, adjustment of the treatment effect estimate used for the decision rule may be omitted when also optimizing the threshold value for the decision rule: this only leads to larger values for ${HR}_{go}^{\ast }$ (i.e., more liberal decision rules) which compensate the adjusted (more conservative) treatment effect estimates. For the same reason, program set-ups $S\left({\hat{\theta}}_2^{\lambda },{\hat{\theta}}_2^u\right)$ and $S\left({\hat{\theta}}_2^{\alpha_{CI}},{\hat{\theta}}_2^u\right)$ (i.e., multiplicative or additive adjustment used for the decision rule and no adjustment applied for the calculation of the number of events for phase III) are not considered. Furthermore, as adjustment of the treatment effect estimate used for the decision rule may be omitted when also optimizing over the threshold value for the decision rule, we did not consider program set-ups where different adjustment parameters used for the decision rule and the calculation of the expected number of events are optimized (in our notation $S\left({\hat{\theta}}_2^{\lambda_1},{\hat{\theta}}_2^{\lambda_2}\right)$ and $S\left({\hat{\theta}}_2^{{\alpha_{CI}}_1},{\hat{\theta}}_2^{{\alpha_{CI}}_2}\right)$).

## Conclusions

Based on our results, we highly recommend using (multiplicatively) adjusted phase II treatment effect estimates for calculation of the phase III number of events in a phase II/III drug development program with go/no-go decision rule (compare Chuang-Stein & Kirby [14], Kirby et al. [15] and De Martini [4, 25]). However, as our results also show that the optimal design parameters of each method depend on the cost and benefit parameters as well as on the applied prior distribution, no general rule exists. In contrast, the design parameters should be determined by applying our proposed optimization procedure for specific values of the parameters in the respective drug development program. Therefore, we provide an user friendly R Shiny App (bias) and an R package (drugdevelopR including the R function optimal_bias) open-source (both assessable via [1]).

## Supplementary information

**Additional file 1. **In the Additional file 1, an overview of formulas in program set-ups $S\left({\hat{\theta}}_2^{s_1},,,{\hat{\theta}}_2^{s_2}\right)$*,s*_1_, *s*_2_ = *λ*, *a*_*CI*_, *u* (A0) and investigation of an alternative definition of program success is given (A1). Furthermore, more details and results of the application example when modelling different population structures in phase II and III (A2), when using a predefined minimal clinically relevant effect for phase III planning (A3), when using a budget constraint (A4), when skipping phase II (A5) and when using a linear function for modelling the gain (A6) are presented. The file Code.R includes the main function calls for generating the datasets and tables, using the R package drugdevelopR.

## Data Availability

The datasets used can be generated with the help of the R package drugdevelopR and the code containing the respective function calls is provided in the additional files (see file Code.R).

## Abbreviations

*α*_*CI*_*,λ*: Adjustment parameter for additive and multiplicative adjustment method, respectively
*bs*: Benefit scenario
*CI*: Confidence interval
*d*_2_*,d*_3_*,d*: Total number of events for phase II, III and the program, respectively
*HR*: True assumed hazard ratio
*κ*: Threshold value for the go/no-go decision rule, *κ = −* log (*HR*_*go*_)
*s*_*1*_*,s*_2_: Estimate used for go/no-go decision and calculation of number of events, respectively
*θ*: True assumed treatment effect, *θ = −* log (*HR*)
