# Supplementary material for: Optimal designs for phase II/III drug development programs including methods for discounting of phase II results
Source: BMC Med Res Methodol. 2020 Oct 9;20:253. doi: 10.1186/s12874-020-01093-w (PMC7547445; doi:10.1186/s12874-020-01093-w)
Supplement: Supplementary file 1 — Additional file 1. In the Additional file 1, an overview of formulas in program set-ups $S\left({\hat{\theta}}_2^{s_1},,,{\hat{\theta}}_2^{s_2}\right)$Sθ^2s1,θ^2s2,s1, s2 = λ, aCI, u (A0) and investigation of an alternative definition of program success is given (A1). Furthermore, more details and results of the application example when modelling different population structures in phase II and III (A2), when using a predefined minimal clinically relevant effect for phase III planning (A3), when using a budget constraint (A4), when skipping phase II (A5) and when using a linear function for modelling the gain (A6) are presented. The file Code.R includes the main function calls for generating the datasets and tables, using the R package drugdevelopR. [file 12874_2020_1093_MOESM1_ESM.docx]

**Additional file 1**

**A0: Overview of formulas in program set-ups** $\boldsymbol{S}\boldsymbol{(}{\hat{\boldsymbol{\theta}}}_{\boldsymbol{2}}^{\boldsymbol{s}_{\boldsymbol{1}}}\boldsymbol{,}{\hat{\boldsymbol{\theta}}}_{\boldsymbol{2}}^{\boldsymbol{s}_{\boldsymbol{2}}}\boldsymbol{)}$**,** ${\boldsymbol{s}_{\boldsymbol{1}}\boldsymbol{, s}}_{\boldsymbol{2}}\boldsymbol{= \lambda,}\boldsymbol{\alpha}_{\boldsymbol{CI}}\boldsymbol{, u}$

| Program set-up | $s_{1}$ | Probability to go to phase III $P$ | $s_{2}$ | Expectation of estimate used for phase III number of events calculation $E$ | Expected number of events for phase III $D$ | Probability of a successful program $S$, where $t_{3}$ is a realization of $T_{3}\vert\hat{\theta}_{2},\theta\sim N(\theta/\sqrt{4/D_{3}},1)$, where ${D_{3}=D}_{3}\left( \hat{\theta}_{2}^{s_{2}} \right)$ |
| --- | --- | --- | --- | --- | --- | --- |
| $S(\hat{\theta}_{2}^{u}, \hat{\theta}_{2}^{u})$ | $u$ | $\Phi\left( \frac{\theta-\kappa}{\sqrt{4/d_{2}}} \right)$ | $u$ | $\frac{1}{P}\int_{\kappa}^{\infty} \hat{\theta}_{2}\cdot f\left( \hat{\theta}_{2}\vert\theta\right)d\hat{\theta}_{2}$ | $\int_{\kappa}^{\infty} \frac{4\cdot\left( z_{1-\alpha}+z_{1-\beta} \right)^{2}}{\hat{\theta}_{2}^{2}}\cdot f\left( \hat{\theta}_{2}\vert\theta\right)d\hat{\theta}_{2}$ | $\int_{z_{1-\alpha}}^{\infty} \int_{\kappa}^{\infty} f\left( t_{3}\vert\hat{\theta}_{2},\theta\right)\cdot f\left( \hat{\theta}_{2}\vert\theta\right)d\hat{\theta}_{2}dt_{3}$ |
| $S(\hat{\theta}_{2}^{u}, \hat{\theta}_{2}^{\lambda})$ |  |  | $\lambda$ | $\frac{1}{P}\int_{\kappa}^{\infty} \lambda\cdot\hat{\theta}_{2}\cdot f\left( \hat{\theta}_{2}\vert\theta\right)d\hat{\theta}_{2}$ | $\int_{\kappa}^{\infty} \frac{4\cdot\left( z_{1-\alpha}+z_{1-\beta} \right)^{2}}{\left( \lambda\cdot\hat{\theta}_{2} \right)^{2}}\cdot f\left( \hat{\theta}_{2}\vert\theta\right)d\hat{\theta}_{2}$ |  |
| $S(\hat{\theta}_{2}^{u}, \hat{\theta}_{2}^{\alpha_{CI}})$ |  |  | $\alpha_{CI}$ | $\frac{1}{P}\int_{\kappa}^{\infty} (\hat{\theta}_{2}- z_{1-\alpha_{CI}}\cdot\sqrt{4/d_{2}})\cdot f\left( \hat{\theta}_{2}\vert\theta\right)d\hat{\theta}_{2}$ | $\int_{\kappa}^{\infty} \frac{4\cdot\left( z_{1-\alpha}+z_{1-\beta} \right)^{2}}{\left( \hat{\theta}_{2}- z_{1-\alpha_{CI}}\cdot\sqrt{4/d_{2}} \right)^{2}}\cdot f\left( \hat{\theta}_{2}\vert\theta\right)d\hat{\theta}_{2}$ |  |
| $S(\hat{\theta}_{2}^{\lambda}, \hat{\theta}_{2}^{\lambda})$ | $\lambda$ | $\Phi\left( \frac{\theta-\frac{\kappa}{\lambda}}{\sqrt{4/d_{2}}} \right)$ | $\lambda$ | $\frac{1}{P}\int_{\frac{\kappa}{\lambda}}^{\infty} \lambda\cdot\hat{\theta}_{2}\cdot f\left( \hat{\theta}_{2}\vert\theta\right)d\hat{\theta}_{2}$ | $\int_{\frac{\kappa}{\lambda}}^{\infty} \frac{4\cdot\left( z_{1-\alpha}+z_{1-\beta} \right)^{2}}{\left( \lambda\cdot\hat{\theta}_{2} \right)^{2}}\cdot f\left( \hat{\theta}_{2}\vert\theta\right)d\hat{\theta}_{2}$ | $\int_{z_{1-\alpha}}^{\infty} \int_{\frac{\kappa}{\lambda}}^{\infty} f\left( t_{3}\vert\hat{\theta}_{2},\theta\right)\cdot f\left( \hat{\theta}_{2}\vert\theta\right)d\hat{\theta}_{2}dt_{3}$ |
| $S(\hat{\theta}_{2}^{\alpha_{CI}}, \hat{\theta}_{2}^{\alpha_{CI}})$ | $\alpha_{CI}$ | $\Phi\left( \frac{\theta-\kappa^{{+\alpha}_{CI}}}{\sqrt{4/d_{2}}} \right)$ | $\alpha_{CI}$ | $\frac{1}{P}\int_{\kappa^{{+\alpha}_{CI}}}^{\infty} \left( \hat{\theta}_{2}- z_{1-\alpha_{CI}}\cdot\sqrt{4/d_{2}} \right)\cdot f\left( \hat{\theta}_{2}\vert\theta\right)d\hat{\theta}_{2}$ | $\int_{\kappa^{{+\alpha}_{CI}}}^{\infty} \frac{4\cdot\left( z_{1-\alpha}+z_{1-\beta} \right)^{2}}{\left( \hat{\theta}_{2}- z_{1-\alpha_{CI}}\cdot\sqrt{4/d_{2}} \right)^{2}}\cdot f\left( \hat{\theta}_{2}\vert\theta\right)d\hat{\theta}_{2}$ | $\int_{z_{1-\alpha}}^{\infty} \int_{\kappa^{{+\alpha}_{CI}}}^{\infty} f\left( t_{3}\vert\hat{\theta}_{2},\theta\right)\cdot f\left( \hat{\theta}_{2}\vert\theta\right)d\hat{\theta}_{2}dt_{3}$ |

**Table A0**: **Overview of formulas in program set-ups** $\boldsymbol{S}\boldsymbol{(}{\hat{\boldsymbol{\theta}}}_{\boldsymbol{2}}^{\boldsymbol{s}_{\boldsymbol{1}}}\boldsymbol{,}{\hat{\boldsymbol{\theta}}}_{\boldsymbol{2}}^{\boldsymbol{s}_{\boldsymbol{2}}}\boldsymbol{)}$, with different estimates selected for the go/no-go decision criterion $\hat{\theta}_{2}^{s_{1}}\geq\kappa$ and for the number of events calculation $D_{3}\left( \hat{\theta}_{2}^{s_{2}} \right)$, ${s_{1}, s}_{2}= \lambda, \alpha_{CI}, u$, where $\hat{\theta}_{2}^{u}= \hat{\theta}_{2}$, $\hat{\theta}_{2}^{\lambda}=\lambda\cdot\hat{\theta}_{2}$ and $\hat{\theta}_{2}^{\alpha_{CI}}= \hat{\theta}_{2}- z_{1-\alpha_{CI}}\cdot\sqrt{4/d_{2}}$ is the unadjusted, multiplicatively adjusted and additively adjusted treatment effect estimate of phase II, respectively. Shown are corresponding expressions ($P, E, D, S$) for calculating the expected probability to go to phase III $p_{go}\left( \hat{\theta}_{2}^{s_{1}} \right)=\int_{-\infty}^{\infty} P\cdot f\left( \theta\right)d\theta$, the expected estimate used for sample size calculation $e_{2}\left( \hat{\theta}_{2}^{s_{1}}, \hat{\theta}_{2}^{s_{2}} \right)= \int_{-\infty}^{\infty} E\cdot f\left( \theta\right)d\theta$, the expected number of events for phase III $d_{3}\left( \hat{\theta}_{2}^{s_{1}}, \hat{\theta}_{2}^{s_{2}} \right)= \int_{-\infty}^{\infty} D\cdot f\left( \theta\right)d\theta$, and the expected probability of a successful program $PsP\left( \hat{\theta}_{2}^{s_{1}}, \hat{\theta}_{2}^{s_{2}} \right)=\int_{-\infty}^{\infty} S\cdot f\left( \theta\right)d\theta$ for a fixed effect $\theta$. Note that $\hat{\theta}_{2}| \theta\sim N(\theta,4/d_{2})$ and $\Phi\left( . \right)$ denotes the distribution function of the standard normal distribution. The adjustment can be “transferred” to $\kappa$, i.e., $\lambda\cdot\hat{\theta}_{2}=\hat{\theta}_{2}^{\lambda}\geq\kappa\Leftrightarrow\hat{\theta}_{2}\geq\kappa/\lambda$ as well as $\hat{\theta}_{2}- z_{1-\alpha_{CI}}\cdot\sqrt{4/d_{2}}=\hat{\theta}_{2}^{\alpha_{CI}}\geq\kappa\Leftrightarrow\hat{\theta}_{2}\geq\kappa+z_{1-\alpha_{CI}}\cdot\sqrt{4/d_{2}}=\kappa^{{+\alpha}_{CI}}$.

**A1:Definition of probability of (true) success**

Recap the definition of the probability of a successful program, which is defined by the probability of the joint event of going to phase III and achieving a significant result in phase III:

$$P\left( \hat{\theta}_{2}\geq\kappa\cap T_{3}>z_{1-\alpha} | \theta\right)=\int_{\kappa}^{\infty} \int_{z_{1-\alpha}}^{\infty} f\left( t_{3}|\hat{\theta}_{2},\theta\right) \cdot f\left( \hat{\theta}_{2}|\theta\right) dt_{3} d\hat{\theta}_{2}.$$

Here, the density of the distribution of the respective argument is indicated by *f*(.) and $t_{3}$ is a realization of the test statistic in phase III $T_{3}|\hat{\theta}_{2},\theta$, where the null hypothesis $H_{0}:\theta\leq0$ is tested against $H_{1}:\theta>0$. Taking the expectation with respect to $\theta$ leads to the expected probability of a successful program which is a statement about a future outcome $t_{3}$ taking the uncertainty in a future but previous outcome $\hat{\theta}_{2}$ and the true parameter $\theta$ into account. Therefore, from Bayesian perspective, it is a predictive probability. However, from a frequentist perspective the prior density *f*(.) covers the null hypothesis $H_{0}$ as well as the alternative space $H_{1}$. Thus, rewriting yields the expected probability of a successful program

$$PsP=\int_{H_{0}} \int_{\kappa}^{\infty} \int_{z_{1-\alpha}}^{\infty} f\left( t_{3}|\hat{\theta}_{2},\theta\right) \cdot f\left( \hat{\theta}_{2}|\theta\right) \cdot f\left( \theta\right) dt_{3} d\hat{\theta}_{2}d\theta+ \int_{H_{1}} \int_{\kappa}^{\infty} \int_{z_{1-\alpha}}^{\infty} f\left( t_{3}|\hat{\theta}_{2},\theta\right) \cdot f\left( \hat{\theta}_{2}|\theta\right) \cdot f\left( \theta\right) dt_{3} d\hat{\theta}_{2}d\theta,$$

where the first summand represents the expected probability of a false positive and the second one the expected probability of a true positive result. Counting the event of a type I error as success of the program may seem counterintuitive. However, it should be noted that this concept reflects that such events occur in practice. Alternatively, one could define the expected probability of a *truly* successful program, i.e.,

$$PtsP=\int_{H_{1}} \int_{\kappa}^{\infty} \int_{z_{1-\alpha}}^{\infty} f\left( t_{3}|\hat{\theta}_{2},\theta\right) \cdot f\left( \hat{\theta}_{2}|\theta\right) \cdot f\left( \theta\right) dt_{3} d\hat{\theta}_{2}d\theta.$$

In general, $PsP\geq PtsP$, with equality if the support of $f\left( \theta\right)$ lays entirely inside of the alternative hypothesis $H_{1}$ and inequality if the support of $f\left( \theta\right)$ includes domains in $H_{0}$. However, if more support of $f\left( \theta\right)$ lays in $H_{0}$, the probability of $T_{3}>z_{1-\alpha}$ decreases and, by construction, the probability of a type I error is small ($=\alpha$). Therefore, the numerical difference of $PsP$ and $PtsP$ can expected to be rather small. Indeed, when comparing the $PsP$ and $PtsP$ in the optimal designs, the difference is smaller than ${10}^{-4}$ or ${10}^{-5}$, respectively (compare Table A1). Furthermore, when using $PtsP$ instead of $PsP$ in the optimization procedure, it has no impact on the optimal design (results not shown).

If “success of a program” only covers correct decisions, then the success definition should also include correct stops of inefficient treatment after phase II like in Götte et al. (2015) (here „Success of a programme is [usually] understood as a statistically significant outcome for the primary endpoint in favour of the experimental treatment in the phase III trial if the drug is efficacious and a stop after phase II in case of an inefficacious drug.“). Consequently, for a utility function, this would require including a penalty if a highly efficacious treatment is stopped after phase II. However, that would be a shift from a fully monetary utility function to a stakeholder perspective utility function as, for example, proposed in Graf et al. (2015). This, however, is not the aim of our paper.

Graf AC, Posch M, König F. Adaptive designs for subpopulation analysis optimizing utility functions. Biometrical Journal 57 (2015) 1, 76–89 DOI: 10.1002/bimj.20130025

**A2: Modelling different population structures in phase II and III**

Modelling different population structures in phase II and III within our framework is implemented by assuming different distributions for the assumed true treatment effect in phase II and III, so that $\hat{\theta}_{2}| \theta_{2} \sim N(\theta_{2},4/d_{2})$ and $T_{3}|\hat{\theta}_{2},\theta_{2}, \theta_{3}\sim N(\theta_{3}/\sqrt{4/D_{3}}, 1)$, where $\theta_{2}≁\theta_{3}$. Thus, the expected (with respect to $\theta_{2}, \theta_{3}, \hat{\theta}_{2}$ and $T_{3}$) probability of a successful program is given by $E[PsP\left( \hat{\theta}_{2}^{s_{1}}, \hat{\theta}_{2}^{s_{2}} \right)]=\int_{-\infty}^{\infty} \int_{-\infty}^{\infty} \int_{z_{1-\alpha}}^{\infty} \int_{-\infty}^{\infty} 1_{\hat{\theta}_{2}^{s_{1}}\geq\kappa}\cdot f\left( t_{3}|\hat{\theta}_{2},\theta_{2}, \theta_{3} \right)\cdot f\left( \hat{\theta}_{2}|\theta_{2} \right)\cdot f\left( \theta_{2} \right)\cdot f\left( \theta_{3} \right)d\hat{\theta}_{2}dt_{3}d\theta_{2}d\theta_{3}$, where $t_{3}$ is a realization of $T_{3}$ (for the other formulas see Table A0 with insertion of $\theta_{2}$ for $\theta$). To investigate the impact of different distributions for the treatment effect in phase II and III, we consider the oncology trial example introduced in the main part and set $\theta_{2}\sim w \cdot N\left( -log\left( 0.69 \right), \left( 4/{210} \right) \right)+ \left( 1 - w \right)\cdot N\left( -log\left( 0.88 \right), \left( 4/{420} \right) \right)$, for $w=0.3, 0.6, 0.9$ and $\theta_{3}\sim\theta_{2}+\gamma$, where $\gamma\in\left\{ -0.025, -0.0125, 0.0125, 0.025 \right\}$ to represent a more pessimistic or more optimistic view about the true treatment effect in phase III compared to phase II. A change of $\pm0.0125$ and $\pm0.025$ for the treatment effect $\theta=-log(HR)$, results in a change of about $\mp0.01$ and $\mp0.02$ for the associated $HR$ in these scenarios (compare Figure A2 and/or investigate the prior distribution by our R Shiny App prior assessable via <https://web.imbi.uni-heidelberg.de/prior/>), respectively. Due to the fact that phase III trials are often conducted in populations that are more heterogeneous than that of the preceding phase II trials, leading to a “dilution” of the treatment effect (Kirby et al., 2012), the scenarios with $\gamma<0$ might be more realistic. However, for completeness, the results of scenarios with $\gamma>0$ are also presented. By comparing the results (presented in Table A2) to the results, where the same distribution is used for the true treatment effect in phase II and III (i.e., $\gamma=0$, compare Table 2), we can quantify how the optimal design parameters and program characteristics change with different views about the true treatment effect in phase II and III.


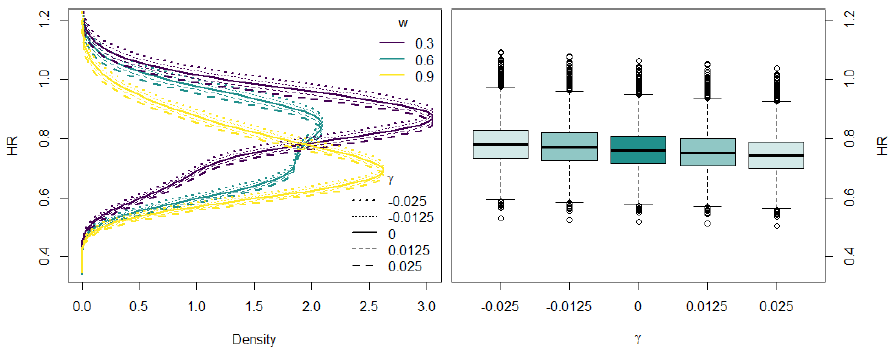
**Figure A2:** Graphical illustration of the distributions used when modeling the true treatment effect in phase II and III differently. Density (for $w=0.3, 0.6, 0.9$) and boxplots (for $w=0.6$) of 10000 samples of the mixture distribution used for modeling the true treatment effect in phase II $\theta_{2}\sim w \cdot N\left( -log\left( 0.69 \right), \left( 4/{210} \right) \right)+ \left( 1 - w \right)\cdot N\left( -log\left( 0.88 \right), \left( 4/{420} \right) \right)$ and III $\theta_{3}\sim\theta_{2}+\gamma$.

**A3: Predefined minimal clinically relevant effect for phase III planning**

In Table A3 the results of the optimization are presented, where the threshold value for the go/no-go decision is only optimized over $\kappa\in\left\{ -\log\left( 0.8 \right), -\log\left( 0.79 \right), \ldots, -log(0.7) \right\}$. The lower bound of the optimization set for $\kappa$ represents the smallest size of treatment effect observed in phase II allowing to go to phase III. By choosing such a “strict” lower bound results in phase II/III programs, where the phase III trials are only conducted if the treatment effect observed is at least $-\log\left( 0.8 \right)$, representing, for example, a minimal clinically relevant effect size. However, the results of this optimization (with constraint on the decision rule parameter) are not optimal with respect to the maximal expected utility (and probability of a successful program) (compare Table A3 and Table 2 and 3).

**A4: Budget constraint**

In Table A4 the results of the optimization with a budget constraint of $K=1000$ (in $\${10}^{5}$) (i.e., $E\left[ c\left( d_{2}, \kappa,s_{2} \right) \right]\leq K$) for different benefit scenarios are presented (if the constraint of $K=1000$ (in $\${10}^{5}$) does not influence the optimization, the results are omitted here). Comparing the restricted settings (compare Table A4) to the unrestricted settings (compare Table 2 and Table 3), we see, that the threshold values for the decision rule, the probability to go to phase III and for a successful program and the number of events tend to be lower in the optimal designs, as well as, the maximal expected utility. The (multiplicative) adjustment method/methods are superior to the unadjusted setting with respect to the maximal expected utility, even though the budget is limited.

**A5: Option to skip phase II**

Setting $d_{2}=0$, $c_{02}=c_{2}=0$ and $p_{go}=1$ reflects the option to skip phase II. The treatment effect used for phase III planning could then be the median of the prior distribution for the true treatment effect $\theta_{plan}=median(f(\theta))$. Therefore, $D_{3}=\frac{4 \cdot\left( z_{1-\alpha}+z_{1-\beta} \right)^{2}}{\left( \theta_{plan} \right)^{2}}s given by nmption of proportional hazards based on asymptotic properties of the log-rank statistics, ficient manner”become av$ and $E\left[ PsP\left( \hat{\theta}_{2}^{s_{1}}, \hat{\theta}_{2}^{s_{2}} \right) \right]=\int_{-\infty}^{\infty} \int_{z_{1-\alpha}}^{\infty} f\left( t_{3}|\theta\right)\cdot f\left( \theta\right)dt_{3}d\theta$, where $t_{3}$ is a realization of $T_{3}|\theta\sim N(\theta/\sqrt{4/D_{3}}, 1)$.

Table A5 shows the program characteristics when skipping phase III. If skipping phase II is the optimal option with respect to *u* and *d* (i.e., higher maximal expected utility and lower number of events, compare scenarios marked with ✓ in Table A5 to respective scenarios in the unadjusted setting $S(\hat{\theta}_{2}^{u}, \hat{\theta}_{2}^{u})$, Table 2), the investment decreases, as no phase II is conducted and lower number of events are needed as compared to conducting a phase II/III program. The success probability, which is the probability of the joint event of going to phase III (here it is always proceeded to phase III, i.e., $p_{go}=1$) and having a significant result in phase III, increases. Thus, if skipping phase II is the optimal option, the expected gain is also higher. In case skipping phase II is the optimal option with respect to *u* only (compare scenarios marked with $ in Table A5), the maximal expected utility is higher, but also higher number of events are needed as compared to conducting a phase II/III program. For scenarios marked with 🗶 in Table A5, skipping phase II is not the optimal option, as the maximal expected utility is lower and the sample size is higher as compared to conducting a phase II/III program. The expected probability of a successful program is quite high. However, it should be noted, that it represents the weighted (by the prior distribution) power of the phase III trial planned with ${HR}_{plan}$ here. Except in scenarios with pessimistic prior and low benefit, skipping phase II is the optimal choice in terms of the utility function/ expected gain. However, somehow surprisingly, it is often not optimal in terms of expected total number of events (only with very optimistic prior or extreme benefit). Thus, in terms of patients exposed to either the experimental or control treatment, skipping phase II is not recommended. Further research would be needed to investigate whether a futility interim analysis in phase III could lead to a compromise between conducting or skipping phase II followed by a phase III without interim analysis.

**A6: Definition of continuous benefit function**

The choice of three effect size categories (and therefore the benefit function) in this paper is based on a report of the German Institute for Quality and Efficiency in Health Care (IQWiG, 2016). However, the proposed framework could also be applied to an alternative set-up. For example, a reviewer asked to make the benefit function a continuous variable. Here, a proportional relationship between benefit and effect size would be considered. In the notation presented in this paper, this translates to a benefit function of the following form

$$g\left( d_{2},\kappa,s_{2} \right)=1_{\left\{ \hat{\theta}_{2}^{s_{1}}\geq\kappa\right\}}\cdot1_{\left\{ T_{3}>z_{1-\alpha} \right\}}\cdot b\cdot T_{3}.$$

However, note the following. The continuous function would be a monotonic increasing function of $-log(HR)$ with limits defined by the $HR$ corresponding to a significant result (i.e., p-value$=0.025$) and an upper limit/plateau for the benefit (at some point further improvement in $HR$ would not lead to further benefit). Figure A6 illustrates the usefulness of the three group approach as a simple to implement and valid approach for the benefit function. Otherwise, complicated non-linear functions need to be evaluated, which do not reflect reality as payers also rather think in categories than non-linear functions.


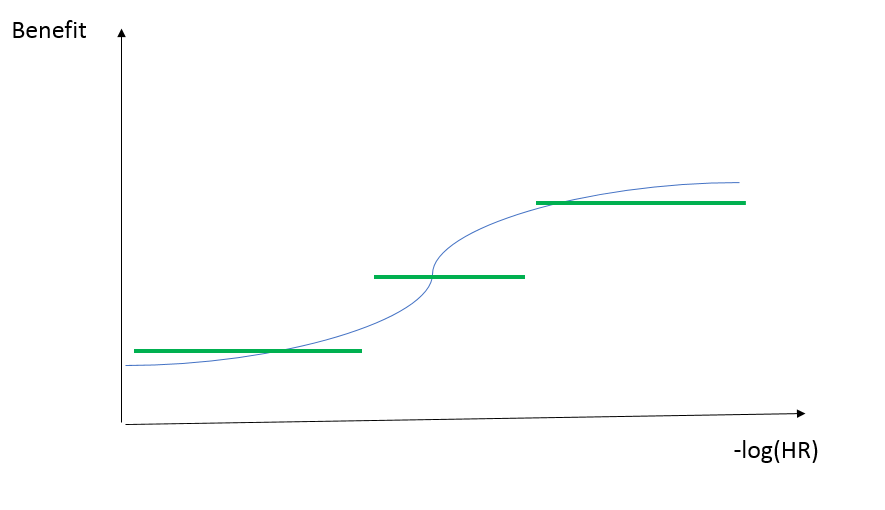


**Figure A6: Illustration of three group approach for the benefit function.**

**Table A1 Numerical results of PsP and PtsP in the optimal designs.** Expected probability of a successful program $PsP$, expected probability of a truly successful program $PtsP$ and their difference$\Delta=PsP-PtsP$ for Program set-ups $S\left( \hat{\theta}_{2}^{u}, \hat{\theta}_{2}^{u} \right), S(\hat{\theta}_{2}^{u}, \hat{\theta}_{2}^{\lambda})$ and $S(\hat{\theta}_{2}^{u}, \hat{\theta}_{2}^{\alpha_{CI}})$, benefit scenarios *bs* 1-7 and weights for the prior distribution *w* = 0.3, 0.6, 0.9.

| *bs* | Program set-up $S(\hat{\theta}_{2}^{u}, \hat{\theta}_{2}^{u})$ | | | Program set-up $S(\hat{\theta}_{2}^{u}, \hat{\theta}_{2}^{\lambda})$ | | | Program set-up $S(\hat{\theta}_{2}^{u}, \hat{\theta}_{2}^{\alpha_{CI}})$ | | |
| --- | --- | --- | --- | --- | --- | --- | --- | --- | --- |
|  | $PsP$ | $PtsP$ | $\Delta$ | $PsP$ | $PtsP$ | $\Delta$ | $PsP$ | $PtsP$ | $\Delta$ |
| $w=0.3$ | | | | | | | | | |
| 1 | 0.242814 | 0.242714 | 0.000100 | 0.253172 | 0.253109 | 0.000063 | 0.237306 | 0.237230 | 0.000076 |
| 2 | 0.283004 | 0.282917 | 0.000088 | 0.290832 | 0.290791 | 0.000041 | 0.274560 | 0.274502 | 0.000058 |
| 3 | 0.308328 | 0.308250 | 0.000078 | 0.330493 | 0.330444 | 0.000049 | 0.305085 | 0.305030 | 0.000056 |
| 4 | 0.327665 | 0.327586 | 0.000078 | 0.350387 | 0.350351 | 0.000036 | 0.324301 | 0.324255 | 0.000046 |
| 5 | 0.349893 | 0.349816 | 0.000077 | 0.376691 | 0.376661 | 0.000030 | 0.337973 | 0.337939 | 0.000034 |
| 6 | 0.354244 | 0.354173 | 0.000072 | 0.378209 | 0.378168 | 0.000041 | 0.359086 | 0.359040 | 0.000046 |
| 7 | 0.377848 | 0.377778 | 0.000070 | 0.395423 | 0.395388 | 0.000034 | 0.369784 | 0.369744 | 0.000040 |
| $w=0.6$ | | | | | | | | | |
| 1 | 0.426987 | 0.426945 | 0.000042 | 0.448837 | 0.448808 | 0.000029 | 0.433207 | 0.433173 | 0.000034 |
| 2 | 0.464128 | 0.464082 | 0.000046 | 0.502358 | 0.502339 | 0.000019 | 0.460127 | 0.460106 | 0.000021 |
| 3 | 0.496407 | 0.496367 | 0.000040 | 0.530838 | 0.530816 | 0.000022 | 0.488986 | 0.488955 | 0.000031 |
| 4 | 0.516983 | 0.516942 | 0.000041 | 0.556385 | 0.556367 | 0.000018 | 0.510748 | 0.510730 | 0.000018 |
| 5 | 0.522593 | 0.522555 | 0.000038 | 0.571658 | 0.571644 | 0.000014 | 0.528559 | 0.528549 | 0.000009 |
| 6 | 0.525210 | 0.525174 | 0.000037 | 0.567929 | 0.567910 | 0.000019 | 0.544729 | 0.544708 | 0.000020 |
| 7 | 0.539637 | 0.539596 | 0.000041 | 0.587339 | 0.587324 | 0.000015 | 0.555276 | 0.555263 | 0.000013 |
| $w=0.9$ | | | | | | | | | |
| 1 | 0.604216 | 0.604202 | 0.000014 | 0.641725 | 0.641717 | 0.000008 | 0.611239 | 0.611229 | 0.000009 |
| 2 | 0.647801 | 0.647787 | 0.000014 | 0.704483 | 0.704477 | 0.000006 | 0.652536 | 0.652529 | 0.000007 |
| 3 | 0.660273 | 0.660261 | 0.000013 | 0.719036 | 0.719029 | 0.000007 | 0.675174 | 0.675165 | 0.000008 |
| 4 | 0.678470 | 0.678457 | 0.000013 | 0.745545 | 0.745540 | 0.000005 | 0.692215 | 0.692210 | 0.000005 |
| 5 | 0.695303 | 0.695289 | 0.000014 | 0.766254 | 0.766250 | 0.000004 | 0.708974 | 0.708971 | 0.000003 |
| 6 | 0.698914 | 0.698901 | 0.000013 | 0.761762 | 0.761757 | 0.000005 | 0.708793 | 0.708788 | 0.000005 |
| 7 | 0.702636 | 0.702624 | 0.000013 | 0.788883 | 0.788878 | 0.000005 | 0.720207 | 0.720203 | 0.000003 |

**Table A5 Overview of scenarios where phase II could be skipped.** Expected utility $u$, expected probability of a successful program $sP$, expected number of events for the program $d$, where the effect size to be used for phase III planning is $\theta_{plan}=median( w \cdot N\left( -log\left( 0.69 \right), \left( 4/{210} \right) \right)+ \left( 1 - w \right)\cdot N\left( -log\left( 0.88 \right), \left( 4/{420} \right) \right))$, for $c_{3}=1$,$c_{03}=150$ in $${10}^{5}$, $\xi_{3}=0.7$ , $1 - \beta= 0.9$, $\alpha= 0.025$ (one sided), benefit scenarios *bs* 1-7 and weights for the prior distribution *w* = 0.3, 0.6, 0.9 when skipping phase II. The column *skip* indicates whether the expected utility is higher and the number of events is lower when skipping phase II compared to proceeding with the phase II/III program (in the unadjusted setting $S(\hat{\theta}_{2}^{u}, \hat{\theta}_{2}^{u})$, compare Table 2) and therefore, whether phase II could be skipped (✓: optimal with respect to $u$ and $d$; $: optimal with respect to $u$) or not (🗶) as optimal option. ${{HR}_{plan}=exp(-\theta}_{plan})$.

| *bs* | $\boldsymbol{w=0.3}$ | | | | | $\boldsymbol{w=0.6}$ | | | | | $\boldsymbol{w=0.9}$ | | | | | |
| --- | --- | --- | --- | --- | --- | --- | --- | --- | --- | --- | --- | --- | --- | --- | --- | --- |
|  | ${HR}_{plan}$ | $d$ | *sP* | $u$ | $skip$ | ${HR}_{plan}$ | $d$ | *sP* | $u$ | $skip$ | ${HR}_{plan}$ | | $d$ | *sP* | $u$ | $skip$ |
| 1 | 0.82 | 1051 | 0.65 | -205 | 🗶 | 0.76 | 577 | 0.70 | 715 | $ | 0.70 | 344 | | 0.76 | 1247 | ✓ |
| 2 | 0.82 | 1051 | 0.65 | 73 | 🗶 | 0.76 | 577 | 0.70 | 1106 | $ | 0.70 | 344 | | 0.76 | 1695 | ✓ |
| 3 | 0.82 | 1051 | 0.65 | 312 | $ | 0.76 | 577 | 0.70 | 1315 | $ | 0.70 | 344 | | 0.76 | 1923 | ✓ |
| 4 | 0.82 | 1051 | 0.65 | 590 | $ | 0.76 | 577 | 0.70 | 1706 | $ | 0.70 | 344 | | 0.76 | 2371 | ✓ |
| 5 | 0.82 | 1051 | 0.65 | 868 | $ | 0.76 | 577 | 0.70 | 2097 | $ | 0.70 | 344 | | 0.76 | 2820 | ✓ |
| 6 | 0.82 | 1051 | 0.65 | 829 | $ | 0.76 | 577 | 0.70 | 1915 | $ | 0.70 | 344 | | 0.76 | 2599 | ✓ |
| 7 | 0.82 | 1051 | 0.65 | 1107 | $ | 0.76 | 577 | 0.70 | 2306 | ✓ | 0.70 | 344 | | 0.76 | 3047 | ✓ |

**Table A2** **Optimal designs for program set-ups with different population structures.** Optimal design parameters $d_{2}^{*}$ and ${HR}_{go}^{*}$, expected utility $u^{*}$, expected number of events in phase III $d_{3}^{*}$, expected probability to go to phase III $p_{go}^{*}$, and expected probability of a successful program ${sP}^{*}$ for the optimal design, for$c_{2}=0.75, c_{3}=1$, $c_{02}=100, c_{03}=150$ in $${10}^{5}$, $\xi_{2}=\xi_{3}=0.7$ , $1 - \beta= 0.9$, $\alpha= 0.025$ (one sided), benefit scenarios *bs* 1, 4, 7, weights for the prior distribution *w* = 0.3, 0.6, 0.9, for the unadjusted program set-up $S(\hat{\theta}_{2}^{u}, \hat{\theta}_{2}^{u})$, for different assumed distributions of the true treatment effect in phase II and III $\theta_{2}≁\theta_{3}\sim\theta_{2}+\gamma$.

|  | $\gamma=-0.025$ | | | | | | $\gamma=-0.0125$ | | | | | | $\gamma=0.0125$ | | | | | | $\gamma=0.025$ | | | | | |
| --- | --- | --- | --- | --- | --- | --- | --- | --- | --- | --- | --- | --- | --- | --- | --- | --- | --- | --- | --- | --- | --- | --- | --- | --- |
| *bs* | ${HR}_{go}^{*}$ | $d_{2}^{*}$ | $d_{3}^{*}$ | $p_{go}^{*}$ | ${sP}^{*}$ | $u^{*}$ | ${HR}_{go}^{*}$ | $d_{2}^{*}$ | $d_{3}^{*}$ | $p_{go}^{*}$ | ${sP}^{*}$ | $u^{*}$ | ${HR}_{go}^{*}$ | $d_{2}^{*}$ | $d_{3}^{*}$ | $p_{go}^{*}$ | ${sP}^{*}$ | $u^{*}$ | ${HR}_{go}^{*}$ | $d_{2}^{*}$ | $d_{3}^{*}$ | $p_{go}^{*}$ | ${sP}^{*}$ | $u^{*}$ |
| $w=0.3$ | | | | | | | | | | | | | | | | | | | | | | | | |
| 1 | 0.78 | 77 | 117 | 0.42 | 0.20 | 17 | 0.79 | 84 | 132 | 0.44 | 0.22 | 46 | 0.81 | 88 | 164 | 0.47 | 0.27 | 110 | 0.81 | 98 | 167 | 0.47 | 0.29 | 145 |
| 4 | 0.83 | 123 | 216 | 0.51 | 0.27 | 298 | 0.83 | 140 | 220 | 0.51 | 0.30 | 362 | 0.85 | 161 | 284 | 0.55 | 0.37 | 506 | 0.85 | 172 | 287 | 0.55 | 0.39 | 585 |
| 7 | 0.84 | 161 | 252 | 0.53 | 0.30 | 523 | 0.85 | 172 | 287 | 0.55 | 0.34 | 614 | 0.86 | 210 | 335 | 0.57 | 0.40 | 816 | 0.87 | 214 | 381 | 0.60 | 0.44 | 926 |
| $w=0.6$ | | | | | | | | | | | | | | | | | | | | | | | | |
| 1 | 0.81 | 126 | 192 | 0.59 | 0.38 | 279 | 0.82 | 123 | 210 | 0.61 | 0.41 | 324 | 0.83 | 137 | 235 | 0.63 | 0.46 | 417 | 0.83 | 137 | 235 | 0.63 | 0.47 | 464 |
| 4 | 0.85 | 175 | 297 | 0.68 | 0.46 | 825 | 0.85 | 182 | 299 | 0.68 | 0.48 | 918 | 0.86 | 210 | 336 | 0.70 | 0.54 | 1110 | 0.86 | 217 | 338 | 0.70 | 0.56 | 1207 |
| 7 | 0.86 | 210 | 336 | 0.70 | 0.49 | 1215 | 0.86 | 217 | 338 | 0.70 | 0.51 | 1336 | 0.87 | 252 | 381 | 0.72 | 0.57 | 1586 | 0.88 | 256 | 424 | 0.74 | 0.60 | 1713 |
| $w=0.9$ | | | | | | | | | | | | | | | | | | | | | | | | |
| 1 | 0.84 | 137 | 272 | 0.77 | 0.56 | 574 | 0.84 | 154 | 278 | 0.78 | 0.59 | 634 | 0.84 | 175 | 283 | 0.79 | 0.63 | 750 | 0.85 | 168 | 304 | 0.80 | 0.66 | 808 |
| 4 | 0.87 | 196 | 363 | 0.84 | 0.63 | 1389 | 0.87 | 221 | 367 | 0.84 | 0.66 | 1506 | 0.87 | 238 | 370 | 0.85 | 0.70 | 1737 | 0.88 | 245 | 401 | 0.86 | 0.73 | 1851 |
| 7 | 0.88 | 235 | 399 | 0.86 | 0.66 | 1939 | 0.88 | 245 | 401 | 0.86 | 0.68 | 2087 | 0.89 | 273 | 437 | 0.88 | 0.74 | 2376 | 0.89 | 287 | 439 | 0.88 | 0.76 | 2517 |

**Table A4** **Optimal designs for program set-ups with budget constraint** $\boldsymbol{K=1000}$ **(in $**$\boldsymbol{10}^{\boldsymbol{5}}$**).** Optimal design parameters $\lambda^{*}$,${\alpha_{CI}}^{*}$, $d_{2}^{*}$ and ${HR}_{go}^{*}$, expected utility $u^{*}$, expected number of events in phase III $d_{3}^{*}$, expected total number of events of program $d^{*}$, expected probability to go to phase III $p_{go}^{*}$, and expected probability of successful program ${sP}^{*}$ for optimal design, for$c_{2}=0.75, c_{3}=1$, $c_{02}=100, c_{03}=150$ in $${10}^{5}$, $\xi_{2}=\xi_{3}=0.7$ , $1 - \beta= 0.9$, $\alpha= 0.025$ (one sided), benefit scenarios *bs* 3/4-7, weights for prior distribution *w* = 0.6, 0.9, for set-ups $S(\hat{\theta}_{2}^{u}, \hat{\theta}_{2}^{u})$, $S(\hat{\theta}_{2}^{u}, \hat{\theta}_{2}^{\lambda})$ and $S(\hat{\theta}_{2}^{u}, \hat{\theta}_{2}^{\alpha_{CI}})$, respectively.

|  | Program set-up $S(\hat{\theta}_{2}^{u}, \hat{\theta}_{2}^{u})$ | | | | | | | Program set-up $S(\hat{\theta}_{2}^{u}, \hat{\theta}_{2}^{\lambda})$ | | | | | | | | Program set-up $S(\hat{\theta}_{2}^{u}, \hat{\theta}_{2}^{\alpha_{CI}})$ | | | | | | | |
| --- | --- | --- | --- | --- | --- | --- | --- | --- | --- | --- | --- | --- | --- | --- | --- | --- | --- | --- | --- | --- | --- | --- | --- |
| *bs* | ${HR}_{go}^{*}$ | $d_{2}^{*}$ | $d_{3}^{*}$ | $d^{*}$ | $p_{go}^{*}$ | ${sP}^{*}$ | $u^{*}$ | $\lambda^{*}$ | ${HR}_{go}^{*}$ | $d_{2}^{*}$ | $d_{3}^{*}$ | $d^{*}$ | $p_{go}^{*}$ | ${sP}^{*}$ | $u^{*}$ | ${\alpha_{CI}}^{*}$ | ${HR}_{go}^{*}$ | $d_{2}^{*}$ | $d_{3}^{*}$ | $d^{*}$ | $p_{go}^{*}$ | ${sP}^{*}$ | $u^{*}$ |
| $w=0.6$ | | | | | | | | | | | | | | | | | | | | | | | |
| 4 |  |  |  |  |  |  |  | 0.700 | 0.81 | 196 | 416 | 612 | 0.60 | 0.53 | 1170 | 0.350 | 0.82 | 224 | 341 | 565 | 0.62 | 0.51 | 1045 |
| 5 |  |  |  |  |  |  |  | 0.700 | 0.81 | 196 | 416 | 612 | 0.60 | 0.53 | 1500 | 0.275 | 0.81 | 243 | 383 | 626 | 0.60 | 0.52 | 1337 |
| 6 |  |  |  |  |  |  |  | 0.750 | 0.82 | 214 | 404 | 618 | 0.62 | 0.54 | 1334 | 0.400 | 0.84 | 231 | 367 | 598 | 0.66 | 0.53 | 1221 |
| 7 |  |  |  |  |  |  |  | 0.750 | 0.82 | 214 | 404 | 618 | 0.62 | 0.54 | 1656 | 0.350 | 0.83 | 240 | 381 | 621 | 0.65 | 0.53 | 1508 |
| $w=0.9$ | | | | | | | | | | | | | | | | | | | | | | | |
| 3 |  |  |  |  |  |  |  | 0.775 | 0.82 | 187 | 408 | 595 | 0.76 | 0.68 | 1371 | 0.375 | 0.83 | 217 | 376 | 593 | 0.78 | 0.67 | 1271 |
| 4 |  |  |  |  |  |  |  | 0.750 | 0.81 | 194 | 406 | 600 | 0.74 | 0.68 | 1810 | 0.375 | 0.83 | 224 | 375 | 599 | 0.78 | 0.67 | 1665 |
| 5 | 0.87 | 228 | 368 | 596 | 0.85 | 0.68 | 1992 | 0.750 | 0.81 | 194 | 406 | 600 | 0.74 | 0.68 | 2253 | 0.300 | 0.81 | 204 | 398 | 602 | 0.74 | 0.65 | 2064 |
| 6 | 0.87 | 228 | 368 | 596 | 0.85 | 0.68 | 1851 | 0.800 | 0.83 | 182 | 412 | 594 | 0.77 | 0.69 | 1998 | 0.375 | 0.83 | 228 | 375 | 603 | 0.79 | 0.67 | 1873 |
| 7 | 0.87 | 229 | 369 | 598 | 0.85 | 0.68 | 2221 | 0.750 | 0.81 | 194 | 406 | 600 | 0.74 | 0.68 | 2435 | 0.375 | 0.83 | 228 | 375 | 603 | 0.79 | 0.67 | 2267 |

**Table A3** **Optimal designs for program set-ups where** $\boldsymbol{min}\left( \boldsymbol{\kappa} \right)\boldsymbol{= -}\log\left( \boldsymbol{0.8} \right)$**.** Optimal design parameters $\lambda^{*}$,${\alpha_{CI}}^{*}$, $d_{2}^{*}$ and ${HR}_{go}^{*}$, expected utility $u^{*}$, expected number of events in phase III $d_{3}^{*}$, expected total number of events of program $d^{*}$, expected probability to go to phase III $p_{go}^{*}$, and expected probability of successful program ${sP}^{*}$ for optimal design, for$c_{2}=0.75, c_{3}=1$, $c_{02}=100, c_{03}=150$ in $${10}^{5}$, $\xi_{2}=\xi_{3}=0.7$ , $1 - \beta= 0.9$, $\alpha= 0.025$ (one sided), benefit scenarios *bs* 1-7, weights for the prior distribution *w* = 0.3, 0.6, 0.9, for the unadjusted program set-up $S(\hat{\theta}_{2}^{u}, \hat{\theta}_{2}^{u})$, multiplicatively adjusted program set-up$S(\hat{\theta}_{2}^{u}, \hat{\theta}_{2}^{\lambda})$ and additively adjusted program set-up $S(\hat{\theta}_{2}^{u}, \hat{\theta}_{2}^{\alpha_{CI}})$, respectively.

|  | Program set-up $S(\hat{\theta}_{2}^{u}, \hat{\theta}_{2}^{u})$ | | | | | | | Program set-up $S(\hat{\theta}_{2}^{u}, \hat{\theta}_{2}^{\lambda})$ | | | | | | | | Program set-up $S(\hat{\theta}_{2}^{u}, \hat{\theta}_{2}^{\alpha_{CI}})$ | | | | | | | |
| --- | --- | --- | --- | --- | --- | --- | --- | --- | --- | --- | --- | --- | --- | --- | --- | --- | --- | --- | --- | --- | --- | --- | --- |
| *bs* | ${HR}_{go}^{*}$ | $d_{2}^{*}$ | $d_{3}^{*}$ | $d^{*}$ | $p_{go}^{*}$ | ${sP}^{*}$ | $u^{*}$ | $\lambda^{*}$ | ${HR}_{go}^{*}$ | $d_{2}^{*}$ | $d_{3}^{*}$ | $d^{*}$ | $p_{go}^{*}$ | ${sP}^{*}$ | $u^{*}$ | ${\alpha_{CI}}^{*}$ | ${HR}_{go}^{*}$ | $d_{2}^{*}$ | $d_{3}^{*}$ | $d^{*}$ | $p_{go}^{*}$ | ${sP}^{*}$ | $u^{*}$ |
| $w=0.3$ | | | | | | | | | | | | | | | | | | | | | | | |
| 1 | 0.80 | 82 | 146 | 228 | 0.46 | 0.24 | 76 | 0.775 | 0.76 | 91 | 160 | 251 | 0.38 | 0.25 | 99 | 0.425 | 0.77 | 91 | 136 | 227 | 0.40 | 0.23 | 78 |
| 2 | 0.80 | 109 | 152 | 261 | 0.45 | 0.26 | 185 | 0.700 | 0.77 | 116 | 222 | 338 | 0.39 | 0.29 | 235 | 0.400 | 0.79 | 113 | 188 | 301 | 0.43 | 0.27 | 194 |
| 3 | 0.80 | 119 | 153 | 272 | 0.45 | 0.26 | 282 | 0.750 | 0.80 | 133 | 275 | 408 | 0.45 | 0.33 | 343 | 0.400 | 0.80 | 130 | 212 | 342 | 0.45 | 0.29 | 300 |
| 4 | 0.80 | 144 | 156 | 300 | 0.44 | 0.27 | 398 | 0.700 | 0.80 | 158 | 320 | 478 | 0.44 | 0.35 | 509 | 0.350 | 0.80 | 168 | 245 | 413 | 0.44 | 0.32 | 440 |
| 5 | 0.80 | 161 | 157 | 318 | 0.44 | 0.27 | 516 | 0.675 | 0.80 | 189 | 348 | 537 | 0.44 | 0.36 | 690 | 0.300 | 0.80 | 190 | 287 | 477 | 0.44 | 0.33 | 591 |
| 6 | 0.80 | 161 | 157 | 318 | 0.44 | 0.27 | 500 | 0.700 | 0.80 | 168 | 321 | 489 | 0.44 | 0.35 | 632 | 0.325 | 0.80 | 189 | 262 | 451 | 0.44 | 0.33 | 559 |
| 7 | 0.80 | 182 | 158 | 340 | 0.44 | 0.28 | 620 | 0.675 | 0.80 | 189 | 348 | 537 | 0.44 | 0.36 | 815 | 0.300 | 0.80 | 200 | 283 | 483 | 0.43 | 0.33 | 715 |
| $w=0.6$ | | | | | | | | | | | | | | | | | | | | | | | |
| 1 | 0.80 | 133 | 176 | 309 | 0.58 | 0.40 | 361 | 0.775 | 0.79 | 126 | 265 | 391 | 0.55 | 0.45 | 412 | 0.425 | 0.80 | 126 | 219 | 345 | 0.57 | 0.42 | 372 |
| 2 | 0.80 | 151 | 180 | 331 | 0.58 | 0.41 | 569 | 0.725 | 0.80 | 175 | 348 | 523 | 0.58 | 0.50 | 696 | 0.350 | 0.80 | 168 | 278 | 446 | 0.58 | 0.46 | 614 |
| 3 | 0.80 | 182 | 184 | 366 | 0.58 | 0.42 | 711 | 0.725 | 0.80 | 175 | 348 | 523 | 0.58 | 0.50 | 844 | 0.325 | 0.80 | 203 | 294 | 497 | 0.58 | 0.48 | 764 |
| 4 | 0.80 | 210 | 187 | 397 | 0.58 | 0.43 | 928 | 0.675 | 0.80 | 210 | 409 | 619 | 0.58 | 0.53 | 1163 | 0.275 | 0.80 | 238 | 338 | 576 | 0.58 | 0.50 | 1039 |
| 5 | 0.80 | 224 | 188 | 412 | 0.58 | 0.43 | 1150 | 0.650 | 0.80 | 249 | 448 | 697 | 0.58 | 0.54 | 1508 | 0.225 | 0.80 | 274 | 390 | 664 | 0.58 | 0.52 | 1334 |
| 6 | 0.80 | 224 | 188 | 412 | 0.58 | 0.43 | 1076 | 0.700 | 0.80 | 231 | 384 | 615 | 0.58 | 0.52 | 1311 | 0.275 | 0.80 | 238 | 338 | 576 | 0.58 | 0.50 | 1195 |
| 7 | 0.80 | 245 | 189 | 434 | 0.58 | 0.44 | 1300 | 0.650 | 0.80 | 259 | 450 | 709 | 0.58 | 0.54 | 1653 | 0.225 | 0.80 | 280 | 387 | 667 | 0.58 | 0.52 | 1492 |
| $w=0.9$ | | | | | | | | | | | | | | | | | | | | | | | |
| 1 | 0.80 | 165 | 206 | 371 | 0.72 | 0.55 | 660 | 0.775 | 0.80 | 175 | 346 | 521 | 0.72 | 0.64 | 751 | 0.400 | 0.80 | 165 | 266 | 431 | 0.72 | 0.58 | 688 |
| 2 | 0.80 | 196 | 212 | 408 | 0.72 | 0.57 | 970 | 0.700 | 0.80 | 200 | 433 | 633 | 0.72 | 0.68 | 1188 | 0.300 | 0.80 | 214 | 356 | 570 | 0.73 | 0.64 | 1064 |
| 3 | 0.80 | 224 | 216 | 440 | 0.73 | 0.58 | 1157 | 0.725 | 0.80 | 224 | 410 | 634 | 0.73 | 0.68 | 1361 | 0.300 | 0.80 | 239 | 350 | 589 | 0.73 | 0.64 | 1251 |
| 4 | 0.80 | 245 | 219 | 464 | 0.73 | 0.59 | 1478 | 0.675 | 0.80 | 256 | 482 | 738 | 0.74 | 0.70 | 1837 | 0.250 | 0.80 | 287 | 397 | 684 | 0.74 | 0.67 | 1665 |
| 5 | 0.80 | 273 | 222 | 495 | 0.74 | 0.59 | 1804 | 0.625 | 0.80 | 294 | 572 | 866 | 0.74 | 0.72 | 2351 | 0.200 | 0.80 | 298 | 470 | 768 | 0.74 | 0.68 | 2109 |
| 6 | 0.80 | 273 | 222 | 495 | 0.74 | 0.59 | 1673 | 0.700 | 0.80 | 270 | 451 | 721 | 0.74 | 0.70 | 2007 | 0.250 | 0.80 | 287 | 397 | 684 | 0.74 | 0.67 | 1855 |
| 7 | 0.80 | 287 | 223 | 510 | 0.74 | 0.60 | 2001 | 0.650 | 0.80 | 298 | 529 | 827 | 0.74 | 0.72 | 2510 | 0.200 | 0.80 | 300 | 469 | 769 | 0.74 | 0.68 | 2294 |
